# FUS/TLS deficiency causes behavioral and pathological abnormalities distinct from amyotrophic lateral sclerosis

**DOI:** 10.1186/s40478-015-0202-6

**Published:** 2015-04-25

**Authors:** Yoshihiro Kino, Chika Washizu, Masaru Kurosawa, Mizuki Yamada, Haruko Miyazaki, Takumi Akagi, Tsutomu Hashikawa, Hiroshi Doi, Toru Takumi, Geoffrey G Hicks, Nobutaka Hattori, Tomomi Shimogori, Nobuyuki Nukina

**Affiliations:** Department of Neuroscience for Neurodegenerative Disorders, Juntendo University Graduate School of Medicine, Tokyo, Japan; CREST (Core Research for Evolutionary Science and Technology), JST, Saitama, Japan; Laboratory for Structural Neuropathology, Brain Science Institute, RIKEN, Saitama, Japan; Laboratory for Molecular Mechanisms of Thalamus Development, Brain Science Institute, RIKEN, Saitama, Japan; Laboratory for Mental Biology, Brain Science Institute, RIKEN, Saitama, Japan; Research Resource Center, Brain Science Institute, RIKEN, Saitama, Japan; Department of Clinical Neurology and Stroke Medicine, Graduate School of Medicine, Yokohama City University, Yokohama, Japan; Graduate School of Biomedical Sciences, Hiroshima University, Hiroshima, Japan; Manitoba Institute of Cell Biology, University of Manitoba, Winnipeg, Canada; Department of Bioinformatics and Molecular Neuropathology, Meiji Pharmaceutical University, Tokyo, Japan

**Keywords:** ALS, FTLD, Essential tremor, RNA-binding protein

## Abstract

**Introduction:**

FUS/TLS is an RNA-binding protein whose genetic mutations or pathological inclusions are associated with neurological diseases including amyotrophic lateral sclerosis (ALS), frontotemporal lobar degeneration, and essential tremor (ET). It is unclear whether their pathogenesis is mediated by gain or loss of function of FUS/TLS.

**Results:**

Here, we established outbred FUS/TLS knockout mice to clarify the effects of FUS/TLS dysfunction *in vivo*. We obtained homozygous knockout mice that grew into adulthood. Importantly, they did not manifest ALS- or ET-like phenotypes until nearly two years. Instead, they showed distinct histological and behavioral alterations including vacuolation in hippocampus, hyperactivity, and reduction in anxiety-like behavior. Knockout mice showed transcriptome alterations including upregulation of Taf15 and Hnrnpa1, while they have normal morphology of RNA-related granules such as Gems.

**Conclusions:**

Collectively, FUS/TLS depletion causes phenotypes possibly related to neuropsychiatric and neurodegenerative conditions, but distinct from ALS and ET, together with specific alterations in RNA metabolisms.

**Electronic supplementary material:**

The online version of this article (doi:10.1186/s40478-015-0202-6) contains supplementary material, which is available to authorized users.

## Introduction

FUS/TLS is an RNA-binding protein associated with neurological diseases. Mutations in *FUS/TLS* cause familial amyotrophic lateral sclerosis (ALS), in which FUS/TLS protein is found in cytoplasmic inclusions [1,2]. In addition, inclusions containing this protein are also observed in people with sporadic ALS and a subset of individuals with frontotemporal lobar degeneration (FTLD) without FUS/TLS mutations [3,4]. More recently, FUS/TLS mutation was found in a family of hereditary essential tremor (ET) [5]. The pathogenic mechanisms of these diseases remain unclear. Therefore, a major research interest is whether FUS/TLS-linked diseases are caused by gain or loss of function of FUS/TLS. Most FUS/TLS mutations in familial ALS are thought to disrupt a nuclear localization signal in the C-terminus, leading to facilitated formation of cytoplasmic stress granules as well as reduced nuclear function [6,7]. Transgenic mice overexpressing FUS/TLS recapitulate some ALS-like phenotypes [8]. On the other hand, reduction in FUS/TLS leads to abnormality in Gems, nuclear granules marked by SMN1 that is involved in the assembly of small nuclear ribonucleoprotein particles and implicated in a motor neuron disease [9,10]. Primary hippocampal neurons from FUS/TLS-deficient embryos show abnormal dendritic spines [11]. One critical piece of information that is still lacking is the phenotype of adult FUS/TLS knockout (KO) mice, as they die within a day after birth [12].

FUS/TLS regulates RNA metabolism, which includes transcription and post-transcriptional processing such as pre-mRNA splicing and mRNA trafficking, some of which are related to neuronal functions [13-16]. The N-terminal region of FUS/TLS forms reversible amyloid-like assemblies, which can be the molecular basis of the formation of RNA-containing granules, including neuronal granules [17,18]. Moreover, RNA metabolism has been highlighted as a potential common pathogenic pathway of ALS/FTLD, because genetic and pathological abnormalities of another RNA-binding protein, TDP-43, have been found in these diseases, as with FUS/TLS [19]. Though previous studies identified transcriptome changes upon transient depletion of FUS/TLS or those in FUS/TLS deficient embryos [13-16], the effects of long-term FUS/TLS depletion on RNA metabolism have been still unclear.

In this study, we analyzed outbred homozygous FUS/TLS knockout (KO) mice to clarify the effects of FUS/TLS depletion on the central nervous system (CNS) in adults. We found abnormalities of the behavior and brain structure, but not ALS- or ET-like phenotypes, in the KO animals.

## Materials and methods

### Animals

TLS^+/-^ mice [12] were maintained on the C57BL/6 J (B6) background. To obtain homozygous TLS KO mice, B6 TLS^+/-^ mice were crossed with ICR mice. The F1 heterozygote mice were intercrossed. To maximize the survival of KO mice, some of the wild type or heterozygote littermates were removed from the cage. Male mice were used in experiments. All experiments with mice were approved by the Animal Experiment Committee of the RIKEN Brain Science Institute.

### Behavioral analysis

The experimental room was maintained in 12 hr light-12 hr dark periods (light period: 8:00 AM to 8:00 PM). Animals were tested blindly for their genotypes. For details of procedures, see Additional file 1: Supplemental Materials and Methods.

### Tremor analysis

A mouse was placed in a plastic box attached with an accelerometer and allowed to move freely. The motion of the mouse was recorded for 1-5 minutes at a sampling rate of 1 kHz. Motion power percentage (MPP) [20] was calculated as (sum of amplitude at 10 ~ 20 Hz)/(sum of amplitude at 0 ~ 100 Hz) × 100.

### Microarray analysis

Three animals were analyzed for each genotype at 8 weeks. 100 ng of total RNA was processed using WT Expression kit (Ambion) and WT Terminal Labeling kit (Affymetrix) and subjected to Mouse Exon 1.0 ST array (Affymetrix) according to the manufacture’s protocol. Dataset analysis was performed using AltAnalyze [21].

### Quantitative PCR (qPCR)

Quantitative PCR analysis was performed using LightCycler 480 (Roche) and FastStart Universal SYBR Green Master (Roche). We used Gapdh for normalization.

### PCR-based detection of alternative RNA processing

The sequence of primers is listed in Additional file 1: Supplemental Material. PCR products were resolved by agarose gel electrophoresis or 5-20% gradient polyacrylamide gel electrophoresis and stained with ethidium bromide or SYBR Gold (Molecular Probe).

## Results

### Absence of ALS- or ET-like phenotypes in outbred FUS/TLS knockout mice

**Figure 1:**
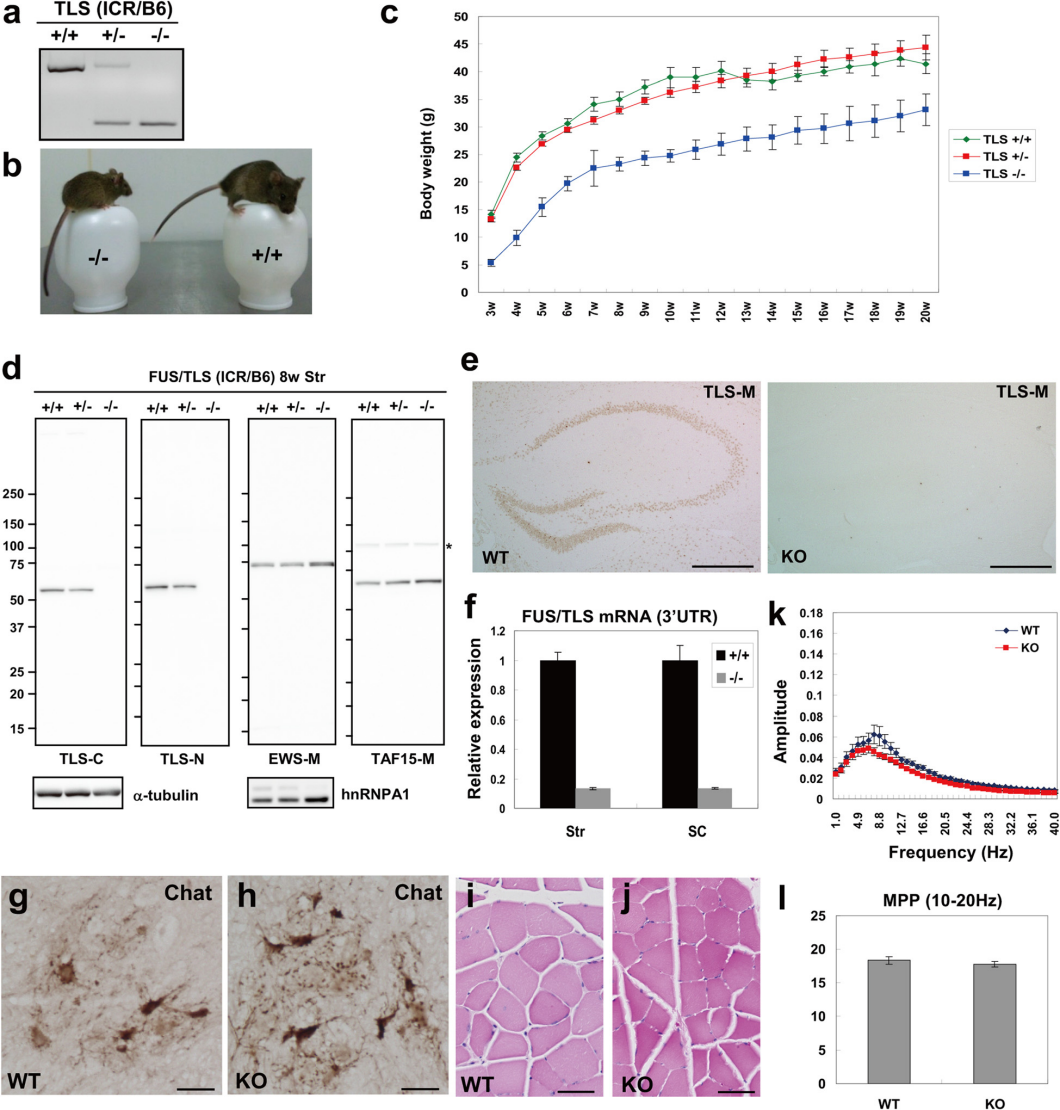
**Phenotypes of outbred homozygote FUS/TLS KO mice. (a)** PCR genotyping of TLS KO mice. **(b)** Photograph of TLS^+/+^ and TLS^-/-^ mice. **(c)** Body weight changes of TLS^-/-^ mice. **(d)** Western blot analysis of TLS protein expression in the striatum of TLS^+/+^, TLS^+/-^, and TLS^-/-^ mice at 8 weeks. Asterisk indicates non-specific cross reactivity of anti-TAF15-M antibody. **(e)** TLS-M immunoreactivity in the brain sections from TLS^+/+^ and TLS^-/-^ mice at 8 weeks. Scale bar: 500 μm. **(f)** qPCR analysis of FUS/TLS mRNA expression in KO mice. The amplicon was located in the 3’UTR (n = 3). **(g, h)** Spinal cord sections of 91-week old WT or KO mouse stained with anti-Chat. Scale bar: 10 μm. **(i, j)** Skeletal muscle sections of 91-week old WT or KO mouse stained with HE. Scale bar: 50 μm. **(k)** Analysis of tremor-like movements of WT or KO mice at 56 weeks. Amplitude at each frequency were determined by fast Fourier transformation of records from an accelerometer (n = 13 for WT, n = 15 for KO). **(l)** Motion power percentage (MPP; see Additional file 1: Supplemental Materials and Methods) of the range of 10-20 Hz were not elevated in FUS/TLS KO mice compared to WT mice (n = 13 for WT, n = 15 for KO). P = 0.32 by a two-tailed unpaired *t*-test. Error bars represent SEM.

Consistent with the previous report [12], we could not obtain homozygous FUS/TLS deficient mice (TLS^-/-^ mice) on the C57BL/6 J (B6) inbred background due to their lethality. There was an independent line of TLS^-/-^ mice that could survive into adulthood on an outbred background, though their CNS phenotypes were not analyzed [22]. Thus, we tried to establish TLS^-/-^ mice on a mixed background of B6 and ICR strains (Figure 1a). Initially, we obtained TLS^-/-^ pups that survived more than one day, but with high mortality until weaning (~80%). In the subsequent cohort, we removed some TLS^+/+^ and TLS^+/-^ littermates from newborn TLS^-/-^ pups, which resulted in the survival of majority of TLS^-/-^ animals after weaning. Thus, the early postnatal mortality of outbred TLS^-/-^ mice could be due to competition with littermates for nutrition rather than to fatal developmental deficits. The body size of TLS^-/-^ animals was smaller than that of TLS^+/+^ and TLS^+/-^ animals (Figure 1b and c). We confirmed FUS/TLS depletion in TLS^-/-^ mice by Western blot, quantitative PCR, and immunohistochemical analyses (Figure 1d-f).

Importantly, the TLS^-/-^ mice did not show apparent motor deficits, even at 90 weeks of age. The number of choline acetyltransferase-positive motor neurons in the spinal cord was not reduced in TLS^-/-^ mice compared to TLS^+/+^ mice (Figure 1g and h). Furthermore, muscle histology of TLS^-/-^ mice did not show apparent atrophic phenotypes (Figure 1i and j). We also measured tremor-like movement in TLS^-/-^ mice. In a control experiment, wild-type mice were treated with harmaline, a widely used model of essential tremor. They showed a clear peak of amplitude at 10-18 Hz (Additional file 1: Figure S1a, Online Resource), reproducing previous results [20]. In contrast, we did not detect any increase in the amplitude at this range in TLS^-/-^ mice compared to TLS^+/+^ mice (Figure 1k and l). We also tested inbred TLS^+/-^ mice on the B6 background but again did not observe tremor-like phenotypes (Additional file 1: Figure S1b, Online Resource). Thus, FUS/TLS depletion was not sufficient for inducing ALS- or ET-like phenotypes *in vivo*.

### Behavioral and histological abnormalities in TLS^-/-^ mice

**Figure 2:**
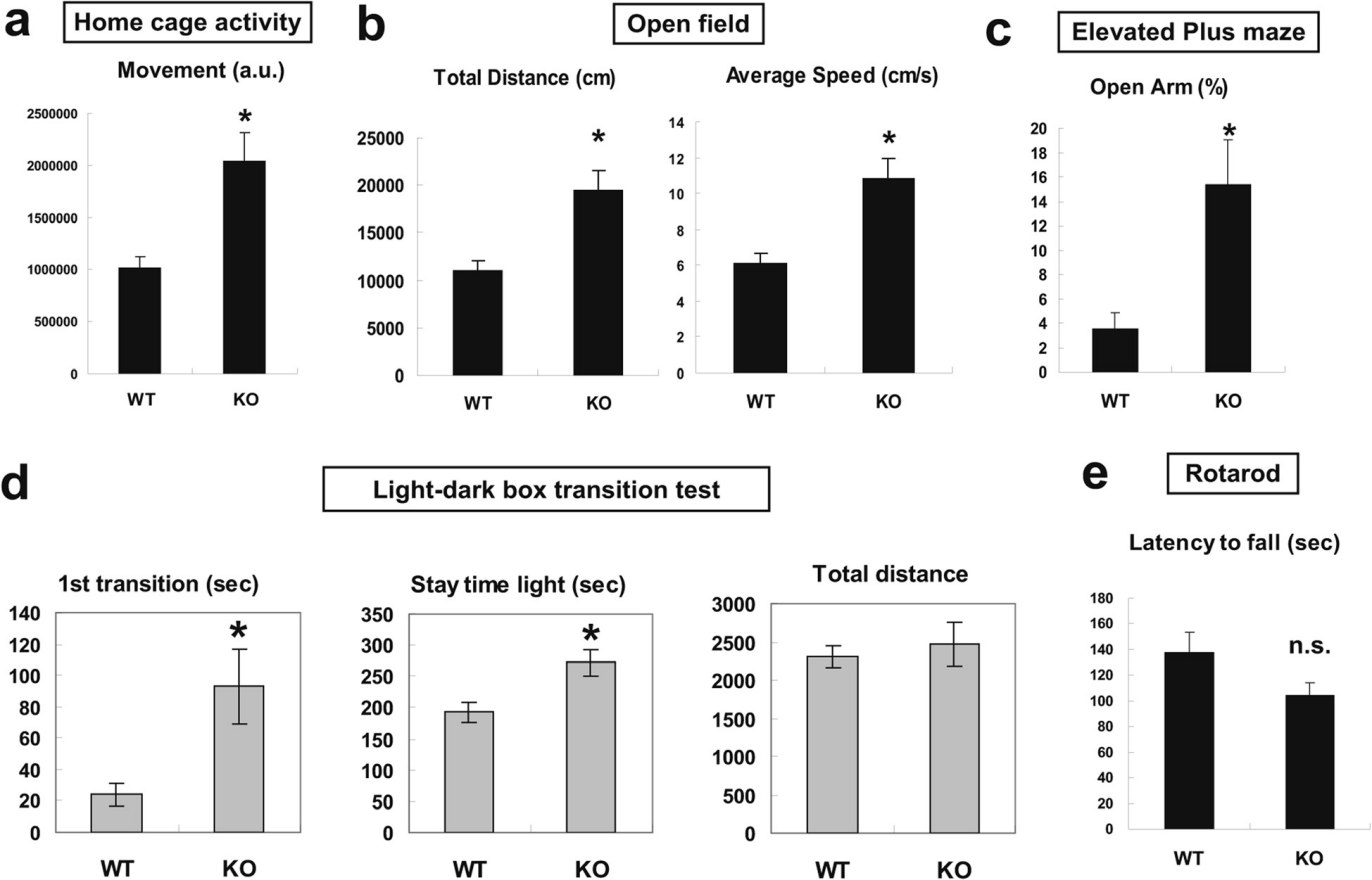
**Behavioral analysis of FUS/TLS KO mice. (a)** Spontaneous home cage activity of WT and TLS KO mice at 8 weeks. Total distance traveled (arbitrary units) in 7 days is shown. P = 0.0020 (n = 12 for WT, n = 11 for KO animals). **(b)** Total distance (P = 0.0062) and average speed of movements (P = 0.0045) of WT and KO animals in an open field test at 34 weeks (n = 12) **(c)** Fraction of time spent in open arms by WT and KO animals in an elevated plus maze test at 35 weeks. P = 0.0059 (unpaired two-tailed *t*-test, n = 12). **(d)** Results of a light-dark transition test of TLS WT and KO mice at 41 weeks. Mean ± SEM (n = 13 for WT, n = 21 for KO). KO mice spent longer time before entering into dark box (P = 0.034) and longer total time in the light box (P = 0.011), whereas their overall movement was not altered (P = 0.67). **(e)** Time spent on an accelerating rotarod by WT and KO animals at 39 weeks (P = 0.073, n = 13 for WT, n = 22 for KO animals). n.s.: not significant. Error bars represent SEM. Statistical significance was evaluated by two-tailed unpaired *t*-test.

To characterize TLS^-/-^ mice, we conducted behavioral analyses. Spontaneous home cage activity was significantly elevated in TLS^-/-^ mice compared to TLS^+/+^ mice in both dark and light periods (Figure 2a and Additional file 1: Figure S2a, b, Online Resource). Hyperactivity of KO mice was also observed in an open field test, as both total distance moved and average speed of KO mice were elevated (Figure 2b). The time course revealed that the locomotive activity of KO mice was indistinguishable from wild-type mice at the beginning but increased as time passed (Additional file 1: Figure S2c, Online Resource), suggesting a habituation-dependent hyperactivity. KO mice exhibited an increase in time spent on open arms in an elevated-plus maze test, indicating a reduction in anxiety-like behavior (Figure 2c). Furthermore, in the light-dark box test, KO animals took longer before entering the dark box and spent more time in the light box, again suggesting reduced anxiety-related behavior (Figure 2d). An accelerated Rotarod test revealed no significant difference in motor performance between WT and KO mice (P = 0.073, Figure 2e). These results demonstrate that FUS/TLS depletion altered some aspects of brain function, while motor function of KO mice was not clearly impaired.

**Figure 3:**
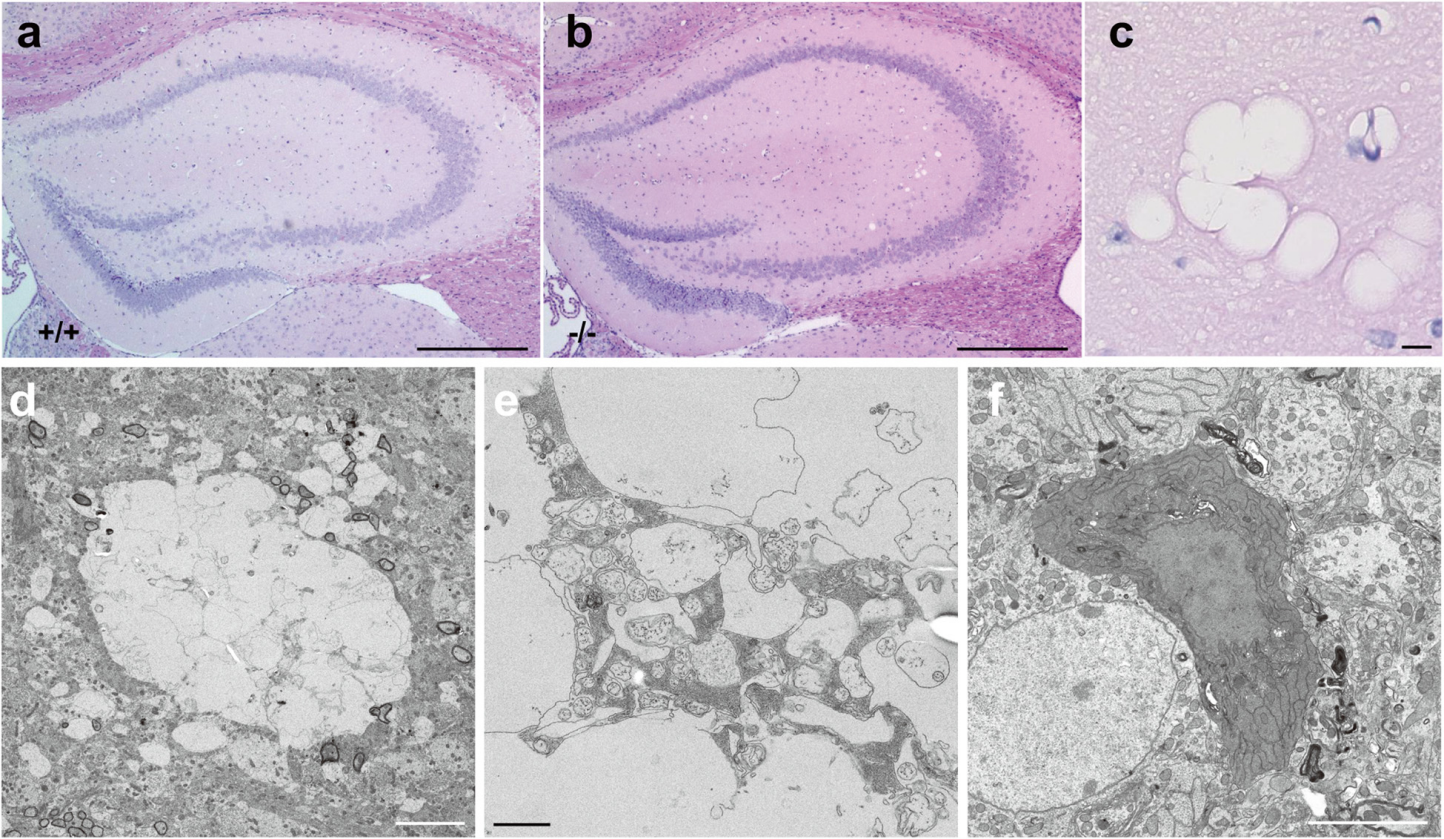
**Histological abnormalities in FUS/TLS KO mice. (a, b)** HE-staining of TLS WT and KO mouse sections at 8 weeks of age. Vacuole-like structures were found in CA3 and the hilus regions of the knockout animal. Scale bar: 500 μm. **(c)** Magnified image of the vacuolar structures in a KO animal. Scale bar: 10 μm. **(d)** Electron microgram of a vacuolar structure in a 12 week-old TLS KO animal. Scale bar: 5 μm. **(e)** Electron microgram of the inside of vacuole-like structure in a KO mice. Scale bar: 1 μm. **(f)** Electron microgram of a dark cell found in a 12 week-old KO animal. Scale bar: 5 μm. Scale bar: 10 μm.

Despite the smaller brain size, the gross structure of the KO brains looked normal. However, we found vacuole-like structures in the hippocampal CA3 and occasionally in the hilus of dentate gyrus of TLS^-/-^ mice (Figure 3a-c). We observed vacuoles in 6 out of 7 KO animals but none (0/7) in WT animals at 8-12 weeks old (P < 0.005, Fisher's exact test). These structures were not observed at 3 or 4 weeks (Additional file 1: Figure S3a, Online Resource). Electron microscopy (EM) revealed that these vacuolar structures contained membranous and organelle-like components (Figure 3d and e). The rim of some vacuolar structures was stained with anti-MAP2, a dendrite marker (Additional file 1: Figure S3b, Online Resource), but not with other antibodies examined. We also found a few darkly stained cells in neuronal cell layers only in TLS^-/-^ mice (Figure 3f). Despite these changes, there was no evidence of increased staining of markers for astrocytes and microglia (Additional file 1: Figure S3c, Online Resource). Thus, TLS^-/-^ mice show unconventional pathological abnormalities in the hippocampus. At later stages, hippocampal vacuolation was observed in 2 out of 3 KO mice at 18 weeks, 1 out of 3 mice at 24 weeks, and 0 out of 3 mice at 47 weeks (Additional file 1: Figure S3d, Online Resource). Thus, the vacuolation phenotype was not apparently progressive. Finally, we did not observe inclusion-like staining of anti-TLS-M in the KO brains at 47 weeks (Additional file 1: Figure S3e, Online Resource).

### Gene expression profile of TLS^-/-^ mice

**Figure 4:**
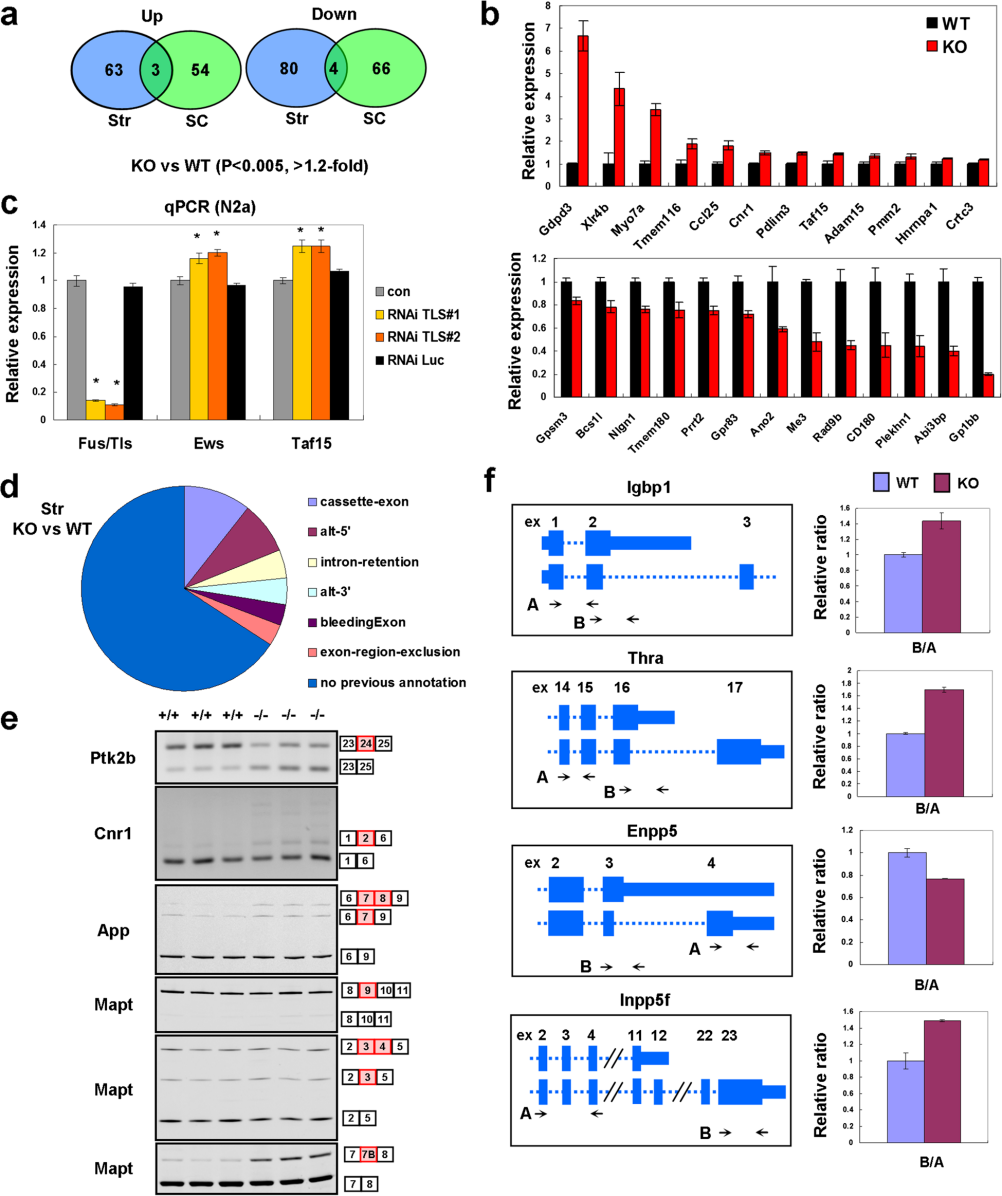
**RNA expression analysis of FUS/TLS KO mice. (a)** Genes up- or down-regulated in the striatum and spinal cord of TLS KO mice (8 weeks old) detected using ExonArray. **(b)** qPCR analysis of up- and down-regulated genes in the striatum of TLS KO mice at 8 weeks (n = 3). All of these genes were significantly down- or up-regulated in the KO mice (p < 0.05, two tailed *t*-test). **(c)** qPCR analysis of FUS/TLS family mRNA in Neuro2A cells depleted of endogenous FUS/TLS using RNAi (n = 4). *P < 0.01, ANOVA followed by Dunnett's test (in comparison with con). Con: untransfected cells. **(d)** Types of alternative RNA processing differentially regulated in the striatum of TLS KO mice. **(e)** Examples of cassette exon splicing in WT and KO mice. Alternative exons are indicated by red boxes. **(f)** Examples of alternative terminal exon splicing. Bar charts show qPCR results using primer sets indicated in the left panels (n = 3). See also Additional file 4: Table S3, online resource.

To gain insight into the genes regulated by FUS/TLS *in vivo*, we conducted an ExonArray analysis using samples from TLS^+/+^ and TLS^-/-^ mice. We tested RNA samples from striatum and spinal cord at 8 weeks old, at which point TLS^-/-^ mice showed behavioral (hyperactivity) and pathological changes (Figures 2 and 3). The expression of >100 genes was altered in either striatum or spinal cord of TLS^-/-^ mice, while the number of overlapping genes in these regions were relatively small (Figure 4a and Additional file 2: Table S1, Online Resource). Thus, a large fraction of altered genes by FUS/TLS depletion were dependent on CNS regions. We did not detect significant enrichment in gene ontology analysis of these datasets. The results of selected genes were confirmed using qPCR (Figure 4b and Additional file 1: Figure S4a, Online Resource). We observed a strong correlation between microarray and qPCR results (r = 0.89, Additional file 1: Figure S5a, see also Additional file 1: Supplemental Materials and Methods, online resource). To validate the expression analysis, we also performed qPCR analysis of additional animal samples that were not used for microarray analysis and found gene expression changes consistent with the initial qPCR results (Additional file 1: Figure S5b, Online Resource). *Gdpd3*, *Taf15* and *Hnrnpa1* were commonly upregulated in the striatum and spinal cord. Taf15 and Ews are homologs of FUS/TLS. We confirmed that knockdown of FUS/TLS up-regulated mRNA expression of *Taf15* and *Ews* in Neuro2a cells (Figure 4c), suggesting a compensatory regulation within the same protein family. Hnrnpa1 is an RNA-binding protein recently implicated in ALS [23]. Protein levels of Taf15, Hnrnpa1 and Ews were elevated in TLS^-/-^ mice (Figure 1d). We did not observe significant expression changes of other genes linked with ALS or FTLD such as *Tarbdp*, *Grn*, *Ubqnl2*, *Vcp*, *Mapt*, *Sod1* or *Optn*. TLS^-/-^ mice also showed upregulation of *Xlr4b* and *Xlr3b* (Figure 4b and Additional file 1: Figure S4a, Online Resource), two related genes implicated in neuronal or behavioral functions [24,25]. Specifically, overexpression of Xlr4b was sufficient for inducing abnormal morphology of dendritic spines [25], a phenotype previously observed in TLS^-/-^ embryonic neurons [11].

### RNA processing and RNA-related granules in TLS^-/-^ mice

We next analyzed RNA processing altered in the TLS^-/-^ mice. Differentially regulated exons included previously known and unknown alternative processing (Figure 4d and Additional file 3: Table S2, Online Resource). We confirmed selected RNA processing alterations using gel-based PCR analysis or qPCR (Additional file 4: Table S3, Online Resource). As examples of differential cassette exon splicing, we confirmed exon inclusion of Ptk2b, Cnr1, App, Ogdh and Gria1 (Figure 4e and Additional file 1: Figure S4d, e, Online Resource). Recent reports suggested that FUS/TLS regulates alternative splicing of Mapt, encoding tau protein [26,27,15,14,16]. FUS/TLS-depleted embryonic brains and primary neurons show elevated inclusion of exon 9, also known as exon 10 in human, related to the "4-repeat" isoforms. We did not observe alteration in exon 9 splicing in our KO mice, as this exon was mostly included even in the WT (Figure 4e). Additionally, we found no significant alteration in the inclusion of exons 3/4 (Figure 4e). However, we observed an increase in the inclusion of exon 7B in KO mice (Figure 4e). This exon consists of 54 nucleotides (Additional file 1: Figure S4f, Online Resource) and its elevation has been reported in another study of FUS/TLS depletion in adult mice [15].

Next, we found changes in alternative usage of terminal exons (Additional file 4: Table S3, Online Resource). Differential expression of transcript isoforms was confirmed for Igbp1, Thra, Enpp5 and Inpp5f (Figure 4f and Additional file 4: Table S3, Online Resource). We also detected exons without previous annotations for RNA processing (Figure 4d). One of such examples was Gdpd3. We found relative down-regulation of 5’ regions of Gdpd3 in KO mice compared to the downstream region (Additional file 1: Figure S4b, Online Resource). 5’-RACE revealed that the 5’ region of Gdpd3 was shorter in TLS^-/-^ mice (Additional file 1: Figure S4c, Online Resource), suggesting a role for FUS/TLS in transcriptional initiation. Collectively, we verified transcriptome changes in TLS^-/-^ mice, consistent with the role of FUS/TLS as a regulator of RNA metabolism.

**Figure 5:**
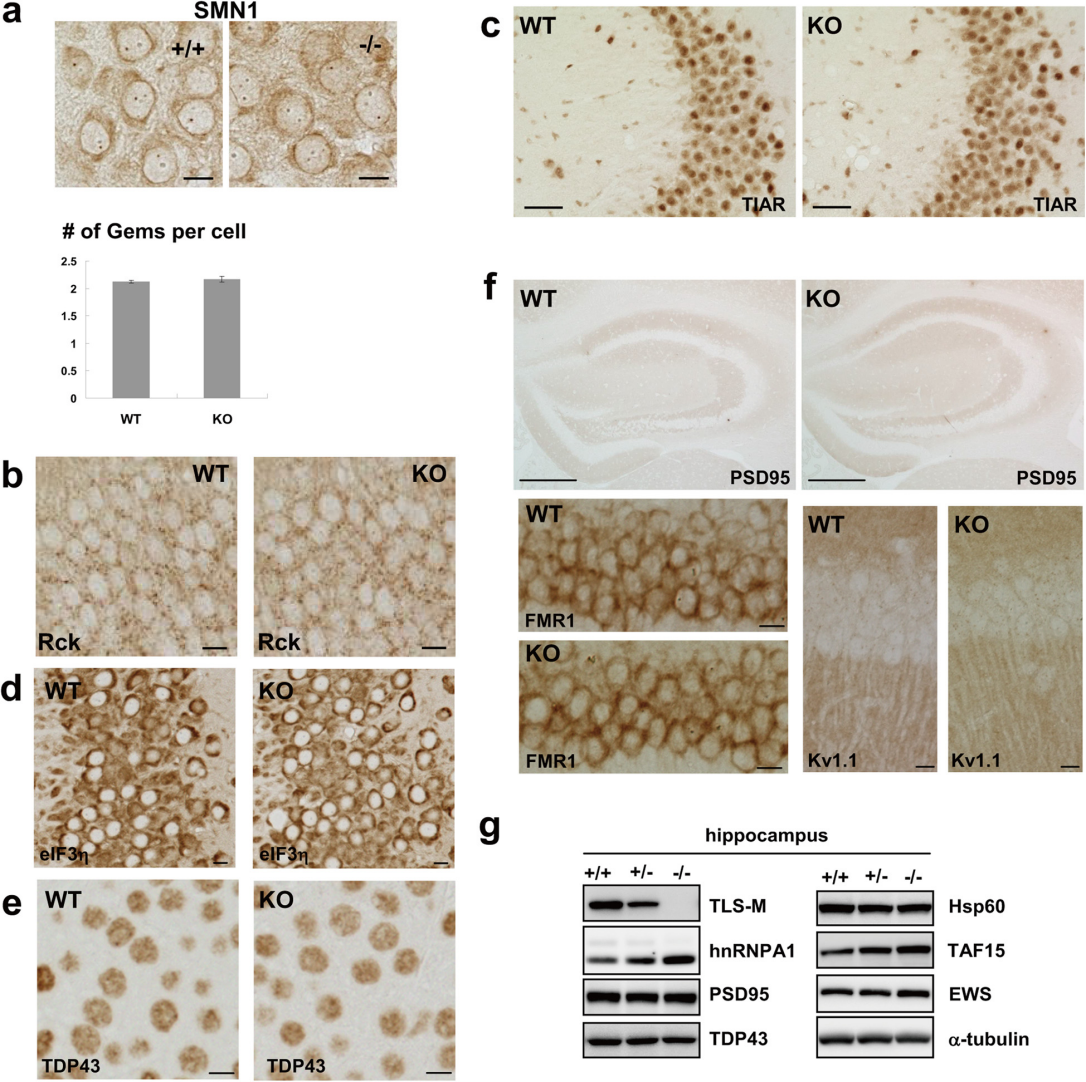
**Protein expression analysis of FUS/TLS KO mice. (a)** Staining of nuclear SMN1 granules (Gems) in the hippocampal CA3 regions of WT and KO mice at 8 weeks. Number of Gems was not altered in KO mice (P = 0.49, n = 3, *t*-test). **(b)** Staining of Rck/Ddx6, a marker of P-body (scale bar: 10 μm). **(c, d)** Immunostaining of stress granules markers (TIAR in **c** and eIF3eta in **d**) in the CA3 region. **(e)** TDP-43 staining in the CA3 region (scale bar 10 μm). **(f)** Staining of PSD-95 in the hippocampus (scale bar: 500 μm), FMR1 and Kv1.1 in the CA1 region (scale bar: 10 μm). **(g)** Protein expression analysis of total lysates from the hippocampus of FUS/TLS knockout mice at 8 weeks.

FUS/TLS has been implicated in the formation of RNA granules and Gems [17,18,10,9]. We observed no overt differences in nuclear SMN1 bodies (Gems) or cytoplasmic P-bodies in TLS^-/-^ mice (Figure 5a and b), suggesting that FUS/TLS is dispensable for these structures. Similarly, staining of stress granule markers as well as TDP-43 did not reveal differences (Figure 5c-e). Since FUS/TLS is implicated in mRNA trafficking [13], we examined protein expression of PSD-95, FMR1, and Kv1.1, as their transcripts are thought to be incorporated into neuronal granules and bound by FUS/TLS [17]. We observed normal expression of these proteins in TLS^-/-^ mice (Figure 5f and g). Thus, depletion of FUS/TLS may not globally affect the integrity of RNA-related granules or the expression of proteins that are locally translated.

## Discussion

Defining FUS/TLS function *in vivo* is essential not only for understanding FUS/TLS-associated diseases but also for designing therapeutic strategies for them. We generated outbred TLS^-/-^ mice, which grew into adulthood. In this TLS^-/-^ strain, truncated FUS/TLS protein has been detected at very low levels in embryonic B cells [12]. We did not detect accumulation of FUS/TLS in TLS^-/-^ mice at 8 and 47 weeks, at the stages when they show phenotypical changes. Considering the aggregation-prone property of FUS/TLS lacking the C-terminus [28], the absence of pathological accumulation of FUS/TLS indicates that the protein expression from the mutant allele was too low to accumulate and supports that the phenotype of TLS^-/-^ mice are mainly attributed to the loss of full-length FUS/TLS protein. Our mice provided several important findings summarized as follows.

(1) KO mice did not show ALS- or ET-like symptoms. The simplest interpretation is that loss of FUS/TLS function is not sufficient to cause ALS or ET, suggesting that FUS/TLS mutations in these diseases must involve some adverse effects distinct from simple loss of function. In line with this, transgenic mice overexpressing wild type FUS/TLS or its N-terminal fragment manifest ALS-like phenotypes together with pathological inclusions [8,28]. We do not rule out the possibility that loss of function of FUS/TLS causes ALS or ET depending on the genetic background or the timing of FUS/TLS depletion. If this is the case, however, our results would provide a therapeutic implication that ALS or ET caused by FUS/TLS depletion can be fully prevented by some genetic factors or compensatory responses, perhaps including upregulation of Taf15 and Ews. It is also possible that these disease are caused by the combinatorial effects of both gain and loss of function of FUS/TLS. (2) KO mice showed behavioral abnormalities, namely hyperactivity and reduced anxiety-related behavior, both of which were supported by multiple different tests. The hyperactivity of TLS^-/-^ mice seemed dependent on habituation, a phenotype observed in human attention deficit/hyperactivity disorder (ADHD) [29]. FUS/TLS is located in a region (16p11.2), where genomic copy number variations were found in ADHD individuals [30]. (3) We also observed vacuole-like structures in the hippocampus of KO mice. Though vacuolation is known in some disease conditions represented by prion diseases, the underlying mechanisms are unclear. As the rim of vacuoles was stained with anti-MAP2, they might originate from dendrites or related structures. There are several genes whose mutation is associated with hippocampal vacuolation in mice (Prnp, Atrn, Mgrn1, Sp4, Gja1, Gjb6, Pnpla6), however, none of them showed altered mRNA expression in FUS/TLS KO mice. Therefore, FUS/TLS might be involved in a novel molecular pathway of vacuolation. Our results suggest that hippocampus is susceptible to FUS/TLS depletion. Though hippocampal vacuolation is not a typical pathological feature of FTLD, loss of FUS/TLS function might contribute to the abnormalities in FLTD hippocampus. In this regard, it is notable that FUS/TLS-positive inclusions in FTLD, but not those in ALS-FUS, are also positive for TAF15 and EWS [31], suggesting composite defects of the FUS/EWS/TAF15 protein family in FTLD-FUS that preclude functional compensation within the family. We noticed that the vacuolation was less frequent at later stages. At present, it is unclear whether the reduced frequency reflected recovery of the phenotype. Interestingly, there is an example of transient CA3 vacuolation, which was induced by Sp4 deficiency [32].

The transcriptome changes detected in our animals are important because some of these changes might be causally or consequentially related to the behavioral and pathological phenotypes. We found that adult TLS^-/-^ mice show alterations in both gene expression and RNA processing, some of which were also identified previously and may represent reliable targets of FUS/TLS (Additional file 5: Table S4, Online Resource). We observed relatively small overlap of genes whose expression levels were altered in CNS regions analyzed (Figure 4a). Consistently, primary neurons from different CNS regions and glial cells show differential changes in their transcriptome when FUS/TLS is depleted [33]. While reduction of Gems was reported in embryonic neurons from an independent strain of FUS/TLS KO mice [9], we observed a normal appearance of Gems. This discrepancy might be due to the cell types, conditions including genetic backgrounds, design of the mutant alleles, developmental stages, and the time span of FUS/TLS depletion in each study. We do not exclude the possibility that the trace amount of truncated FUS/TLS from the mutant allele in our mice prevented the abnormalities of Gems and some other RNA processing, which led to the viability of these mice. In any case, our results indicate that FUS/TLS may not be absolutely required for Gems. In this way, our mice provide unique opportunities to reveal the long-term effects of FUS/TLS depletion in adults.

## Conclusion

In conclusion, our results demonstrate that deficiency of FUS/TLS leads to behavioral and pathological abnormalities that might be relevant to neuropsychiatric or neurodegenerative disorders, including FTLD. However, FUS/TLS KO mice did not develop phenotypes similar to ALS or ET. We also identified transcriptome changes caused by FUS/TLS depletion, which would be useful for future identification of biomarkers as well as molecular pathways reflecting FUS/TLS dysfunction *in vivo*.

## Availability of supporting data

The data sets supporting the results of this article are included within the article and its additional files.

## Additional files

Additional file 1: Figure S1. (a) Analysis of tremor-like movement of wild type mouse with or without harmaline treatment. (b) Analysis of tremor-like movement of inbred (B6) heterozygous FUS/TLS knockout mice and controls. **Figure S2.** (a, b) Spontaneous home cage activity of WT and TLS-/- mice (the same date set as Figure 2a). Distance of movement are shown for each 30 minutes in (a). Movement of mice in dark and light periods were separetely analyzed in (b). (c) Time course of locomotive activity in an open field test of FUS/TLS KO mice. **Figure S3.** (a) Hippocampal sections of 3 week-old animals stained with hematoxylin-eosin. (b) MAP2 immunoreactivity around vacuole-like structures in the KO hippocampus. (c) Immunohistochemical analysis of GFAP and Iba1 in the hippocampus CA3 of TLS KO mice at 8 weeks. (d) Hippocampal sections of KO mice stained with HE. Shown are KO mice with (18 and 24 weeks) and without vacuolation (47 weeks). (e) TLS immunoreactivity in the CA3 region of WT and KO mice at 47 weeks. **Figure S4.** (a) Altered gene expression in the spinal cord of FUS/TLS KO mice at 8 weeks. (b) Analysis of Gdpd3 expression in the striatum of TLS-/- and control mice. (c) Determination of the 5’ end of Gdpd3 mRNA in WT and KO mice by 5’RACE. (d) Alternative splicing of mutually exclusive exons 4 and 5 of Ogdh in TLS KO mice. (e) Alternative splicing of mutually exclusive exons 16 and 17 of Gria1. (f) Gene structure of Mapt, adopted from UCSC mouse genome browser. **Figure S5.** (a) Comparison of ExonArray and qPCR results (n=37 genes). (b) Replication of qPCR results using additional sets of animals. Additional file 2: Table S1. Gene expression analysis of FUS/TLS-deficient mice. Additional file 3: Table S2. RNA processing analysis of FUS/TLS-deficient mice. Additional file 4: Table S3. Summary of validated RNA processing alterations in the TLS KO striatum. Additional file 5: Table S4. Transcriptome changes found in both this study and Lagier-Tourenne et al.

## References

[1] Kwiatkowski TJ, Bosco DA, Leclerc AL, Tamrazian E, Vanderburg CR, Russ C, Davis A, Gilchrist J, Kasarskis EJ, Munsat T, Valdmanis P, Rouleau GA, Hosler BA, Cortelli P, de Jong PJ, Yoshinaga Y, Haines JL, Pericak-Vance MA, Yan J, Ticozzi N, Siddique T, McKenna-Yasek D, Sapp PC, Horvitz HR, Landers JE, Brown RH (2009). Mutations in the FUS/TLS gene on chromosome 16 cause familial amyotrophic lateral sclerosis. Science.

[2] Vance C, Rogelj B, Hortobagyi T, De Vos KJ, Nishimura AL, Sreedharan J, Hu X, Smith B, Ruddy D, Wright P, Ganesalingam J, Williams KL, Tripathi V, Al-Saraj S, Al-Chalabi A, Leigh PN, Blair IP, Nicholson G, de Belleroche J, Gallo JM, Miller CC, Shaw CE (2009). Mutations in FUS, an RNA processing protein, cause familial amyotrophic lateral sclerosis type 6. Science.

[3] Deng HX, Zhai H, Bigio EH, Yan J, Fecto F, Ajroud K, Mishra M, Ajroud-Driss S, Heller S, Sufit R, Siddique N, Mugnaini E, Siddique T (2010) FUS-immunoreactive inclusions are a common feature in sporadic and non-SOD1 familial amyotrophic lateral sclerosis. Ann Neurol 67(6):739–748, doi:10.1002/ana.22051 10.1002/ana.22051 PMC4376270 20517935

[4] Woulfe J, Gray DA, Mackenzie IR (2010) FUS-immunoreactive intranuclear inclusions in neurodegenerative disease. Brain Pathol 20(3)):589–597, doi:10.1111/j.1750-3639 10.1111/j.1750-3639.2009.00337.x PMC8094734 19832837

[5] Merner ND, Girard SL, Catoire H, Bourassa CV, Belzil VV, Riviere JB, Hince P, Levert A, Dionne-Laporte A, Spiegelman D, Noreau A, Diab S, Szuto A, Fournier H, Raelson J, Belouchi M, Panisset M, Cossette P, Dupre N, Bernard G, Chouinard S, Dion PA, Rouleau GA (2012). Exome sequencing identifies FUS mutations as a cause of essential tremor. Am J Hum Genet.

[6] Dormann D, Rodde R, Edbauer D, Bentmann E, Fischer I, Hruscha A, Than ME, Mackenzie IR, Capell A, Schmid B, Neumann M, Haass C (2010) ALS-associated fused in sarcoma (FUS) mutations disrupt Transportin-mediated nuclear import. EMBO J 29(16):2841–2857, doi:10.1038/emboj.2010.143 10.1038/emboj.2010.143 PMC2924641 20606625

[7] Kino Y, Washizu C, Aquilanti E, Okuno M, Kurosawa M, Yamada M, Doi H, Nukina N (2011). Intracellular localization and splicing regulation of FUS/TLS are variably affected by amyotrophic lateral sclerosis-linked mutations. Nucleic Acids Res.

[8] Mitchell JC, McGoldrick P, Vance C, Hortobagyi T, Sreedharan J, Rogelj B, Tudor EL, Smith BN, Klasen C, Miller CC, Cooper JD, Greensmith L, Shaw CE (2013). Overexpression of human wild-type FUS causes progressive motor neuron degeneration in an age- and dose-dependent fashion. Acta Neuropathol.

[9] Tsuiji H, Iguchi Y, Furuya A, Kataoka A, Hatsuta H, Atsuta N, Tanaka F, Hashizume Y, Akatsu H, Murayama S, Sobue G, Yamanaka K (2013). Spliceosome integrity is defective in the motor neuron diseases ALS and SMA. EMBO Mol Med.

[10] Yamazaki T, Chen S, Yu Y, Yan B, Haertlein TC, Carrasco MA, Tapia JC, Zhai B, Das R, Lalancette-Hebert M, Sharma A, Chandran S, Sullivan G, Nishimura AL, Shaw CE, Gygi SP, Shneider NA, Maniatis T, Reed R (2012). FUS-SMN protein interactions link the motor neuron diseases ALS and SMA. Cell Rep.

[11] Fujii R, Okabe S, Urushido T, Inoue K, Yoshimura A, Tachibana T, Nishikawa T, Hicks GG, Takumi T (2005). The RNA binding protein TLS is translocated to dendritic spines by mGluR5 activation and regulates spine morphology. Curr Biol.

[12] Hicks GG, Singh N, Nashabi A, Mai S, Bozek G, Klewes L, Arapovic D, White EK, Koury MJ, Oltz EM, Van Kaer L, Ruley HE (2000). Fus deficiency in mice results in defective B-lymphocyte development and activation, high levels of chromosomal instability and perinatal death. Nat Genet.

[13] Fujii R, Takumi T (2005). TLS facilitates transport of mRNA encoding an actin-stabilizing protein to dendritic spines. J Cell Sci.

[14] Ishigaki S, Masuda A, Fujioka Y, Iguchi Y, Katsuno M, Shibata A, Urano F, Sobue G, Ohno K (2012). Position-dependent FUS-RNA interactions regulate alternative splicing events and transcriptions. Sci Rep.

[15] Lagier-Tourenne C, Polymenidou M, Hutt KR, Vu AQ, Baughn M, Huelga SC, Clutario KM, Ling SC, Liang TY, Mazur C, Wancewicz E, Kim AS, Watt A, Freier S, Hicks GG, Donohue JP, Shiue L, Bennett CF, Ravits J, Cleveland DW, Yeo GW (2012). Divergent roles of ALS-linked proteins FUS/TLS and TDP-43 intersect in processing long pre-mRNAs. Nat Neurosci.

[16] Rogelj B, Easton LE, Bogu GK, Stanton LW, Rot G, Curk T, Zupan B, Sugimoto Y, Modic M, Haberman N, Tollervey J, Fujii R, Takumi T, Shaw CE, Ule J (2012). Widespread binding of FUS along nascent RNA regulates alternative splicing in the brain. Sci Rep.

[17] Han TW, Kato M, Xie S, Wu LC, Mirzaei H, Pei J, Chen M, Xie Y, Allen J, Xiao G, McKnight SL (2012). Cell-free formation of RNA granules: bound RNAs identify features and components of cellular assemblies. Cell.

[18] Kato M, Han TW, Xie S, Shi K, Du X, Wu LC, Mirzaei H, Goldsmith EJ, Longgood J, Pei J, Grishin NV, Frantz DE, Schneider JW, Chen S, Li L, Sawaya MR, Eisenberg D, Tycko R, McKnight SL (2012). Cell-free formation of RNA granules: low complexity sequence domains form dynamic fibers within hydrogels. Cell.

[19] Thomas M, Alegre-Abarrategui J, Wade-Martins R (2013). RNA dysfunction and aggrephagy at the centre of an amyotrophic lateral sclerosis/frontotemporal dementia disease continuum. Brain.

[20] Martin FC, Le Thu A, Handforth A (2005). Harmaline-induced tremor as a potential preclinical screening method for essential tremor medications. Mov Disord.

[21] Emig D, Salomonis N, Baumbach J, Lengauer T, Conklin BR, Albrecht M (2010) AltAnalyze and DomainGraph: analyzing and visualizing exon expression data. Nucleic Acids Res 38:W755–762, doi:10.1093/nar/gkq405 10.1093/nar/gkq405 PMC2896198 20513647

[22] Kuroda M, Sok J, Webb L, Baechtold H, Urano F, Yin Y, Chung P, de Rooij DG, Akhmedov A, Ashley T, Ron D (2000). Male sterility and enhanced radiation sensitivity in TLS(-/-) mice. EMBO J.

[23] Kim HJ, Kim NC, Wang YD, Scarborough EA, Moore J, Diaz Z, MacLea KS, Freibaum B, Li S, Molliex A, Kanagaraj AP, Carter R, Boylan KB, Wojtas AM, Rademakers R, Pinkus JL, Greenberg SA, Trojanowski JQ, Traynor BJ, Smith BN, Topp S, Gkazi AS, Miller J, Shaw CE, Kottlors M, Kirschner J, Pestronk A, Li YR, Ford AF, Gitler AD, Benatar M, King OD, Kimonis VE, Ross ED, Weihl CC, Shorter J, Taylor JP (2013). Mutations in prion-like domains in hnRNPA2B1 and hnRNPA1 cause multisystem proteinopathy and ALS. Nature.

[24] Davies W, Isles A, Smith R, Karunadasa D, Burrmann D, Humby T, Ojarikre O, Biggin C, Skuse D, Burgoyne P, Wilkinson L (2005). Xlr3b is a new imprinted candidate for X-linked parent-of-origin effects on cognitive function in mice. Nat Genet.

[25] Cubelos B, Sebastian-Serrano A, Beccari L, Calcagnotto ME, Cisneros E, Kim S, Dopazo A, Alvarez-Dolado M, Redondo JM, Bovolenta P, Walsh CA, Nieto M (2010) Cux1 and Cux2 regulate dendritic branching, spine morphology, and synapses of the upper layer neurons of the cortex. Neuron 66(4):523–535, doi:10.1016/j.neuron.2010.04.038 10.1016/j.neuron.2010.04.038 PMC2894581 20510857

[26] Orozco D, Tahirovic S, Rentzsch K, Schwenk BM, Haass C, Edbauer D (2012). Loss of fused in sarcoma (FUS) promotes pathological Tau splicing. EMBO Rep.

[27] Goedert M, Spillantini MG (2011). Pathogenesis of the tauopathies. J Mol Neurosci.

[28] Shelkovnikova TA, Peters OM, Deykin AV, Connor-Robson N, Robinson H, Ustyugov AA, Bachurin SO, Ermolkevich TG, Goldman IL, Sadchikova ER, Kovrazhkina EA, Skvortsova VI, Ling SC, Da Cruz S, Parone PA, Buchman VL, Ninkina NN (2013). Fused in sarcoma (FUS) protein lacking nuclear localization signal (NLS) and major RNA binding motifs triggers proteinopathy and severe motor phenotype in transgenic mice. J Biol Chem.

[29] Sagvolden T, Russell VA, Aase H, Johansen EB, Farshbaf M (2005). Rodent models of attention-deficit/hyperactivity disorder. Biol Psychiatry.

[30] Williams NM, Zaharieva I, Martin A, Langley K, Mantripragada K, Fossdal R, Stefansson H, Stefansson K, Magnusson P, Gudmundsson OO, Gustafsson O, Holmans P, Owen MJ, O'Donovan M, Thapar A (2010) Rare chromosomal deletions and duplications in attention-deficit hyperactivity disorder: a genome-wide analysis. Lancet 376(9750):1401–1408, doi:10.1016/S0140-6736(10)61109-9 10.1016/S0140-6736(10)61109-9 PMC2965350 20888040

[31] Neumann M, Bentmann E, Dormann D, Jawaid A, DeJesus-Hernandez M, Ansorge O, Roeber S, Kretzschmar HA, Munoz DG, Kusaka H, Yokota O, Ang LC, Bilbao J, Rademakers R, Haass C, Mackenzie IR (2011). FET proteins TAF15 and EWS are selective markers that distinguish FTLD with FUS pathology from amyotrophic lateral sclerosis with FUS mutations. Brain.

[32] Zhou X, Long JM, Geyer MA, Masliah E, Kelsoe JR, Wynshaw-Boris A, Chien KR (2005). Reduced expression of the Sp4 gene in mice causes deficits in sensorimotor gating and memory associated with hippocampal vacuolization. Mol Psychiatry.

[33] Fujioka Y, Ishigaki S, Masuda A, Iguchi Y, Udagawa T, Watanabe H, Katsuno M, Ohno K, Sobue G (2013). FUS-regulated region- and cell-type-specific transcriptome is associated with cell selectivity in ALS/FTLD. Sci Rep.

