# Supplementary material for: FUS/TLS deficiency causes behavioral and pathological abnormalities distinct from amyotrophic lateral sclerosis
Source: Acta Neuropathol Commun. 2015 Apr 25;3:24. doi: 10.1186/s40478-015-0202-6 (PMC4408580; doi:10.1186/s40478-015-0202-6)
Supplement: Additional file 1: Figure S1. — (a) Analysis of tremor-like movement of wild type mouse with or without harmaline treatment. (b) Analysis of tremor-like movement of inbred (B6) heterozygous FUS/TLS knockout mice and controls. Figure S2. (a, b) Spontaneous home cage activity of WT and TLS-/- mice (the same date set as Figure 2a). Distance of movement are shown for each 30 minutes in (a). Movement of mice in dark and light periods were separetely analyzed in (b). (c) Time course of locomotive activity in an open field test of FUS/TLS KO mice. Figure S3. (a) Hippocampal sections of 3 week-old animals stained with hematoxylin-eosin. (b) MAP2 immunoreactivity around vacuole-like structures in the KO hippocampus. (c) Immunohistochemical analysis of GFAP and Iba1 in the hippocampus CA3 of TLS KO mice at 8 weeks. (d) Hippocampal sections of KO mice stained with HE. Shown are KO mice with (18 and 24 weeks) and without vacuolation (47 weeks). (e) TLS immunoreactivity in the CA3 region of WT and KO mice at 47 weeks. Figure S4. (a) Altered gene expression in the spinal cord of FUS/TLS KO mice at 8 weeks. (b) Analysis of Gdpd3 expression in the striatum of TLS-/- and control mice. (c) Determination of the 5’ end of Gdpd3 mRNA in WT and KO mice by 5’RACE. (d) Alternative splicing of mutually exclusive exons 4 and 5 of Ogdh in TLS KO mice. (e) Alternative splicing of mutually exclusive exons 16 and 17 of Gria1. (f) Gene structure of Mapt, adopted from UCSC mouse genome browser. Figure S5. (a) Comparison of ExonArray and qPCR results (n=37 genes). (b) Replication of qPCR results using additional sets of animals. [file 40478_2015_202_MOESM1_ESM.pdf]

Additional file 1 : Acta Neuropathologica Communications

FUS/TLS deficiency causes behavioral and pathological abnormalities distinct from amyotrophic lateral sclerosis

Yoshihiro Kino, Chika Washizu, Masaru Kurosawa, Mizuki Yamada, Haruko Miyazaki, Takumi Akagi, Tsutomu Hashikawa, Hiroshi Doi, Toru Takumi, Geoffrey Hicks, Nobutaka Hattori, Tomomi Shimogori, and Nobuyuki Nukina.

Contents of Additional file 1:

- **Supplemental Materials and Methods**

- **Supplemental References**

- **Supplemental Figures**

Figure S1 (related to Figure 1): Analysis of tremor-like movement of mice

Figure S2 (related to Figure 2): Behavioral analysis of FUS/TLS knockout mice

Figure S3 (related to Figures 3 and 4): Histological analysis of FUS/TLS knockout mice

Figure S4 (related to Figure 4): Transcriptome analysis of FUS/TLS knockout mice

Figure S5 (related to Figure 4): Experimental validation of RNA analysis

- **Supplemental Tables (separate EXCEL files)**

Additional file 2: Table S1. Gene expression analysis of FUS/TLS-deficient mice

Additional file 3: Table S2. RNA processing analysis of FUS/TLS-deficient mice

Additional file 4: Table S3. Summary of validated RNA processing alterations in the TLS KO striatum

Additional file 5: Table S4. Transcriptome changes found in both this study and Lagier-Tourenne et al.

Supplemental Materials and Methods

cDNA clones and constructs

For making RNAi constructs, oligonucleotides corresponding to an artificial microRNA were inserted into the Esp3I site of R-miR [6]. miTls-253 was made using miTls-253-Fw: 5'-TGCTGAGAAGATTGTGAACTTTGGCTGTTTTGGCCACTGACTGACAGCCAAAGCACAATCTTCT-3' and miTls-253-Rv: 5'-CCTGAGAAGATTGTGCTTTGGCTGTCAGTCAGTGGCCAAAACAGCCAAAGTTCACAATCTTCTC-3'. miTls-853 was made using miTls853-Fw: 5'-TGCTGAATTGTAACATTCTCGCCTAGGTTTTGGCCACTGACTGACCTAGGCGAATGTTACAATT-3' and miTls853-Rv: CCTGAATTGTAACATTCGCCTAGGTCAGTCAGTGGCCAAAACCTAGGCGAGAATGTTACAATTC-3'.

Animals

Heterozygous FUS/TLS ($TLS^{+/-}$) knockout mice [4] were maintained on a C57BL/6J (B6) background. To obtain homozygous TLS knockout mice, heterozygous TLS mice (B6) were crossed with ICR mice. The F1 TLS heterozygote mice on a mixed background of B6 and ICR were intercrossed to obtain outbred $TLS^{-/-}$ mice. For behavioral analysis, *in vitro* fertilization was performed to obtain large numbers of animals. To maximize the survival of homozygote KO mice, roughly half of the pups with normal body size, which were presumably wild type or heterozygotes, were removed from the cages. The genotypes of all animals were confirmed by PCR. In all experiments, only male mice were used for all genotypes. Animals used for life span

analysis were not used in behavioral or histological analyses. All the experiments with mice were approved by the Animal Experiment Committee of the RIKEN Brain Science Institute.

Behavioral analysis

The experimental room was maintained in 12 hr light-12hr dark periods (light period: 8:00 AM to 8:00 PM). We maintained three to five animals per cage, with an exception for ICR/B6 mice with one or two animals per cage to avoid fighting. The number of animals used in an experiment was determined by the capacity and availability of experimental equipments as well as experimenters. Each experiment was conducted by the same experimenter. Animals were tested blindly for genotype. All animals used for experiments were included in data analysis.

A Rotarod test was performed using MK-610 (Muromachi Kikai Tokyo, Japan). The time duration of mice on the Rotarod was measured. In one set of experiments, three trials per animal were performed with intervals of at least 30 minutes between trials. The average of three trials was used for data analysis (two-tailed unpaired t-test). Animals were tested at 39 weeks (n=13 for $TLS^{+/+}$, n=22 for $TLS^{-/-}$).

For the analysis of spontaneous home cage activity, mouse movement was automatically monitored using SCANET (Melquest, Toyama, Japan) for 6 or 7 days. Each animal was maintained in a cage with bedding, water, and food and let move freely. The cage was located inside of an apparatus equipped with an infrared sensor to detect mouse movement. Animals were tested at 8 or 9 weeks of age (n=12 for $TLS^{+/+}$, n=11 for $TLS^{-/-}$).

For an open field test, an automatic detection system with four 50 cm x 50 cm chambers was used for ICR/B6 mice at 36 weeks (n=10 for $TLS^{+/+}$, and n=14 for $TLS^{-/-}$).

In both cases, mouse behavior was analyzed for 30 minutes. Total distance of the movement, moving speed, and time spent in the center area were analyzed. ICR/B6 mice were analyzed.

The elevated plus maze test was performed using equipment from O’ Hara & Co. , Ltd. and Time EP2 software. Animal behavior on the plus maze was recorded for 10 minutes. The time spent on open arms, closed arms, and the central platform was analyzed by the software. ICR/B6 outbred animals were tested at 34 weeks old (n=10 for $TLS^{+/+}$ and n=16 for $TLS^{-/-}$).

Light-dark box analysis was performed using an apparatus previously described [10]. Animals were first put in the light compartment. The time duration of the first transition into dark compartment, the number of transitions, and total time spent in each chamber were analyzed for 10 minutes. ICR/B6 outbred animals were tested at 40 weeks old (n=13 for $TLS^{+/+}$ and n=21 for $TLS^{-/-}$).

Tremor analysis

Tremor measurement was based on a method described previously (Park et al., 2010). A wireless accelerometer, MVP-RF8-EC (MicroStone, Nagano, Japan), was attached to the bottom of a plastic box with a cover that can be hung in the air by a plastic string. A mouse was placed inside of the box and allowed to move freely. The motion of the mouse was recorded by the accelerometer for 1-5 minutes at a sampling rate of 1 kHz. The resultant values of every 1024 data points on the x axis was subjected to fast Fourier transformation using Excel 2003 (Microsoft) and obtained 58 power spectrum data sets per minute. The averaged amplitude at every frequency was calculated. For analysis longer than 1 minute, the averaged values of the data at every minute was presented. We used data corresponding to frequencies ranging from 1 to 100 Hz. Motion

power percentage [8] was calculated as (sum of amplitude at 10~20 Hz)/(sum of amplitude at 0~100 Hz) x 100 for each 1.024 second and averaged for every minute. Animals were tested at 55 weeks old for ICR/B6 $TLS^{+/+}$ (n=13) and $TLS^{-/-}$ (n=15) mice and at 66 weeks old for B6 inbred $TLS^{+/+}$ (n=14) and $TLS^{+/-}$ (n=10) mice. For positive control experiments, either saline or harmaline dissolved in saline (30 mg/kg) was injected intraperitoneally into wild type animals at 8-12 weeks old. Tremor measurements were performed before and after injection for each animal. The results were analyzed by two-tailed unpaired t-test.

Antibodies

Primary antibodies used in this study are listed bellow. Rabbit anti-TLS-M, anti-TLS-C, anti-EWS-M, anti-TAF15-M, and anti-Uqbln2 were described previously [1,2]. Secondary antibodies were anti-mouse, anti-rabbit, anti-goat, or anti-Guinea pig IgG antibodies conjugated to Alexa-488, Alexa-546, or Alexa-634 (Molecular Probe), to horse radish peroxidase (ECL anti-rabbit or anti-mouse IgG Horseradish Peroxidase Linked whole antibody, GE Healthcare, and Peroxidase-conjugated AffiniPure Donkey Anti-Goat IgG, Jackson Immuno research), or to biotin (Vector Laboratories).

Antibody	Source	Number	Host
TLS-M	Doi et al. 2008	-	rabbit
TLS-C	Doi et al. 2008	-	rabbit
EWS-M	Doi et al. 2008	-	rabbit
TAF15-M	Doi et al. 2008	-	rabbit
UBQLN2	Doi et al. 2004	-	rabbit
hnRNP A1 (9H10) :	Santa Cruz	sc-56700	mouse
NMDAR1 (NR1-CT)	upstate	06-311	rabbit
PSD95	Abcam	ab18258	rabbit
DDX5	Abcam	ab10261	goat

alpha-tubulin	Sigma	T9026	mouse
gamma-tubulin (C-20)	Santa Cruz	sc-7396	goat
HSC70 (K-19)	Santa Cruz	sc-1059	goat
Sqstm1	Progen	GP62-C	Guinea pig
Sqstm1	MBL	PM045	rabbit
TDP43	Proteintech Group	BC001487 10782-1	rabbit
TLS 4H11	Santa Cruz	sc-47711	mouse
GFP	Molecular Probe	A6455	rabbit
GFP	Roche	11 814 460 001	mouse
GFP	MBL	598	rabbit
Chat	Millipore	AB144P	goat
Rck/p54	MBL	PD009	rabbit
SMN	BD	610646	mouse
MAP2	Millipore	AB5622	rabbit
GFAP	Dako	Z0334	rabbit
Iba1	Wako	019-19741	rabbit
TIAR (C-18)	Santa Cruz	sc-1749	goat
eIF3η (N-20)	Santa Cruz	sc-16377	goat
RFP	MBL	PM005	rabbit
LaminB (M-20)	Santa Cruz	sc-6217	goat
FUS/TLS	Sigma	HPA008784	rabbit
Ubiquitin	Chemicon	MAB1510	mouse
G3BP	BD transduction	611126	mouse
FMRP	Chemicon	MAB2160	mouse
HSP60(K-19)	Santa Cruz	sc-1722	goat
hnRNPA2B1 (DP3B3)	Santa Cruz	sc-32316	mouse
GRP78 (Bip)	stressgen	SPA-826	rabbit
Bip	BD	610978	mouse
NEUROFILAMENT200	Sigma	N5389	mouse
Neurofilament (DA2)	Abcam	ab7255	Mouse
MBP (SMI-94)	Covance	SMI-94R	mouse
Kv1.1 (K20/78)	NeuroMAB	75-007	mouse

Biochemical analysis of mouse tissues

All extraction buffers were supplemented with 1× complete protease inhibitor cocktail

(Roche) and pre-chilled on ice except for those containing 2% SDS. For preparation of total lysate used for SDS-PAGE or AGERA, frozen brain regions or spinal cord was homogenized in hypotonic buffer [10 mM HEPES (pH7.9), 1.5 mM $MgCl_2$, 10 mM KCl] at a ratio of 1.5 ml buffer per 0.1 mg tissue sample using Teflon homogenizers on ice. NP-40 was then added to the homogenate to a final concentration of 0.5 % and incubated on ice for 30 minutes. The resultant lysates were frozen at -80°C for storage or used immediately for subsequent preparation. 10% SDS and 5 M NaCl were added to the lysate to a final concentration of 2% and 0.35 M, respectively. The mixture was sonicated for 20 seconds twice using a Branson Sonifier at room temperature, and then boiled for 5 minutes. Protein concentration was determined using Micro BCA protein assay (Thermo Scientific). Protein concentration of samples were adjusted using the same buffer (hypotonic buffer + SDS + NaCl). Protein concentration of the adjusted samples was determined using Micro BCA protein assay again. If necessary, protein concentration was further adjusted. Samples were used immediately or frozen at -80°C for storage. Samples were boiled for 5 minutes and subjected to SDS-PAGE using 5-20% gradient gels (e-PAGEL, ATTO) or AGERA. After electrophoresis, proteins were transferred to a PVDF membrane for 90 minutes at 150 mA for 1 h using a semidry blotter at room temperature or at 40 V using a tank blotter in a cold room. Transferred proteins were detected by immunostaining.

Cell death analysis

Cell death in mouse brain sections was analyzed using a Deadend colorimetric TUNEL system (Promega), Fluorojade-C (Millipore), or FD NeuroSilver kit II (FD Neurotechnologies, Inc), according to the manufacturer's recommendation. We examined 8 or 10 week-old mice but did not detect a specific increase in dead or dying

cells in TLS KO animals compared to WT animals.

Cell culture and transfection

Neuro2a (N2a) cells were maintained in Dulbecco's modified Eagle's medium (DMEM) supplemented with 10% fetal bovine serum. For transfection assays, cells were cultured in 12-well plates or 10 cm dishes and transfected with plasmids using Lipofectamine 2000 (Invitrogen) according to the manufacturer's instructions. Cells were harvested at 24-72 h post transfection. For cell sorting, transfected cells in several 10 cm dishes were detached using trypsin and collected in DMEM. For RNA interference-mediated knockdown of endogenous FUS/TLS, miTls-253 and miTls-853 were transfected. miLuc-200 [6] was used as a negative control. For RNA preparation, harvested cells were treated with RNeasy Mini Kit together with RNase-Free DNase Set (QIAGEN).

Microarray analysis

Total RNA was extracted from frozen mouse brain regions or spinal cord using TRIzol reagent (Invitrogen). The resultant RNA was further purified using RNeasy and RNase-free DNase kit (Qiagen). 100 ng of RNA was amplified with Ambion WT Expression kit and labeled with WT Terminal Labeling kit (Affymetrix). Mouse Exon 1.0 ST array (Affymetrix) was hybridized with labeled probes according to the manufacture's protocol. Dataset analysis was performed using GeneSpring GX (Agilent Technologies) and AltAnalyze [3]. For gene expression levels, we noticed correction of multiple comparisons by the method of Benjamini and Hochberg was too stringent. For example, the raw and corrected P values for FUS/TLS in the ExonArray analysis of striatum were 0.001 and 0.62, respectively. Thus, exploration of altered genes using the corrected P-values could result in a large number of false negatives and was not

practically informative. This may be mainly due to the small size of samples (n=3 for each region) and the relatively large number of tests (> 28000 probesets). We compared qPCR and ExonArray results and found that the majority of tested genes showed concordant results (r=0.89, Fig. S5a, left). In many cases, genes with marginal P values (0.01~0.05) in ExonArray could be reproduced by qPCR analysis (Fig. S5a, right). Assuming that qPCR provides accurate measurements of expression, these results indicate an empirical false discovery rate around 15% (4/27) when raw p-value <0.05 was used as a threshold. For counting the number of differentially expressed genes (in Fig. 4a) and for gene ontology analysis, we used a more stringent threshold P<0.005 to select genes. Thus, we adopted the use of raw p values rather than FDR-corrected ones. For the same reason, we used raw P values for the evaluation of RNA processing analysis in AltAnalyze. Using a loosened threshold in the ExonArray analysis was supported by a recent report [11]. We used the FIRMA method [9] for detection of RNA processing. NCBI37/mm9 was used as a mouse genome assembly. Gene ontology analysis was performed using DAVID [5].

Quantitative PCR (qPCR)

Quantitative PCR analysis was performed using LightCycler 480 (Roche) according to the manufacturer's protocol. For quantification using SYBR Green, FastStart Universal SYBR Green Master (Roche Applied Science) was used. Primer sets were designed using either PrimerExpress (Applied Biosystems) or PrimerQuest (Integrated DNA Technologies). Gapdh was used as a reference of normalization. For most qPCR experiments, we used cDNA samples of mice that were also used for microarray analysis. We prepared additional striatal cDNA samples for the experiment shown in Supplementary Fig. S5b. The sequences of primers are listed as follows.

Name	sequence (5'-3')	Name	sequence (5'-3')
RT-Tls-3UTR-Fw	CCCAGTGTTACCCTTGTTATTTTGT	RT-Smarca1-Fw	TCGGGCACATCGCATTG
RT-Tls-3UTR-Rv	CCCCCCAAAAAAAAATATCCA	RT-Smarca1-Rv	GTGTTGTCTGTAATGAGACGGAAGA
RT-Htt-int1-probe	FAM-CAGCTCCCTGTCCCGGCGG-ZEN/IBFQ	RT-Efemp1-Fw	CAATGAACCTGGGAAGTTCTCAT
RT-Gdpd3-Fw	GGGTCAGACCGGCACATG	RT-Efemp1-Rv	GCTGCGCACCACTTCGT
RT-Gdpd3-Rv	CATGGGAGTCCTTGGAAATTTC	RT-Igfbp4-Fw	AACATCCCAACAACAGCTTCAA
RT-Gpdp3-9-Fw	TCTCCTGTACTTTGTTCTGCCCAC	RT-Igfbp4-Rv	TCTAATTTTGGCCATATGCTTCTG
RT-Gpdp3-9-Rv	ACTGCTTCCATGGTGTTCTCCAGT	RT-Tgfbi-Fw	AGGTTGCCCAGCAGCTCTT
RT-Gdpd3-153-Fw	ACTGGAGAACACCATGGAAGCAGT	RT-Tgfbi-Rv	GATCCCACAACTCCCATGGT
RT-Gdpd3-153-Rv	ATCCTTATTTAGGCCTGACTGGCG	RT-Dcn-Fw	GCTGCGGAAATCCGACTTC
RT-Gdpd3-208-Fw	TTGGAATTTGACTGCCAGCTCACC	RT-Dcn-Rv	TGCCGCCCAGTTCTATGAC
RT-Gdpd3-208-Rv	ATCCTTATTTAGGCCTGACTGGCG	RT-Ews-Fw	CAATCCGGGCTGTGGAAA
RT-Gdpd3-379-Fw	GGGTCAGACCGGCACATG	RT-Ews-Rv	GCCTTACACTGGTTGCATTCTG
RT-Gdpd3-379-Rv	CATGGGAGTCCTTGGAAATTTC	RACE-Gdpd3-340Fw	GAGTTGGAAATTTACTTCTCAC
RT-Cort-Fw	TGTCCAAGAGCCAGGAAAGG	RACE-Gdpd3-250Rv	GCCATCCCGGGTGAGCTGGCAG
RT-Cort-Rv	TCTTGCAGGGCTTTTTATCCA	RACE-Gdpd3-280Rv	ACTGGCGGGACAGGTTCTTG
RT-Miip-Fw	AGAGCAGCGGTGTGGAAGA	Ptk2b-spl-ex23-Fw	CCCAGCCGGCCCAAGTACAGACCT
RT-Miip-Rv	GCCGGCGGTTGATACG	Ptk2b-spl-ex25-Rv	CAAGCATCTTTCCTCTCGCCTCAG
RT-Arnt2-Fw	TGGTCCACTGCACAGGTTACA	Cnr1-spl-ex1-Fw	GGCGACAGGTGCCGAGGGAGCTTC
RT-Arnt2-Rv	TTCTTCGGGTATGGTCATTCCT	Cnr1-spl-ex5-Rv	GTCTCCTGCCGTCATCTTTTCTTG
RT-Hrh3-Fw	GCCACTGCTATGCTGAGTTCTTCT	Ogdh-spl-ex3-Fw	AAACACCAATGCTGGAGCCCCACC
RT-Hrh3-Rv	AAGAAGGTAACGCTGAGGAAGGGT	Ogdh-spl-ex7-Rv	CTCCAGCCGACGGATGATCTCCC
RT-Rad9b-Fw	TGTATTTCCAAGACAGCCAGCCCT	Mapt-splicing-ex2-Fw	AAGACCATGCTGGAGATTACACTCTGC
RT-Rad9b-Rv	TGTCAGAAGAGCAATGGCCTCAGT	Mapt-splicing-ex5-Rv	GGTGTCTCCGATGCCTGCTTCTT
RT-Casp9-Fw	TCTTCACGCGCGACATGA	Mapt-splicing-ex89-Fw	TCCCCCTAAGTCACCATCAG
RT-Casp9-Rv	CCGCCGAGACCCAGATC	Mapt-splicing-ex11-Rv	GCCAATCTTCGACTGGACTC
RT-Myo7a-Fw	CGAGGGATTCAGCCTTTTTG	RT-Igbp1-ex1-2Fw	ACTCCTTGAGAAGGCTGCTGGAAT
RT-Myo7a-Rv	CAGTCTGTCAGGTGTCGGACAA	RT-Igbp1-ex1-2Rv	ACTTCAGGTCGGTGGAAGCAATCT
RT-Gabra2-Fw	GCCGAATGTCCCATGCA	RT-Igbp1-ex2-int2Fw	AGCTCCTCCATGGCCTATCCAAAT
RT-Gabra2-Rv	GGGCATGAATGAGCATCCAT	RT-Igbp1-ex2-int2Rv	CACACACACACACACACCACACAA
RT-Igfals-Fw	GGGCCTCAGTCACCTTTGG	RT-Thra-ex16-int16Fw	TACGTCAACCACCGCAAACACAAC
RT-Igfals-Rv	CGTGTCAGGCAGGACCACTA	RT-Thra-ex16-int16Rv	GCACTCGACTTTCATGTGGAGGAA
RT-Zfp69-Fw	TGTCAGTGGCAGGATATCAACTTT	RT-Thra-ex14-15Fw	AGAGGCCTTCAGCGAGTTTACCAA
RT-Zfp69-Rv	GGTCCCTCTCCTTTTTCCAACT	RT-Thra-ex14-15Rv	AGGAGGATGATCTGGTCTTCGCAA
RT-Masp2-Fw	CGCCTGCGCCTCTACTTC	RT-Thra-ex16-17Fw	TACGTCAACCACCGCAAACACAAC
RT-Masp2-Rv	TCATACTCGCAGCGGTAAGAGA	RT-Thra-ex16-17Rv	ACCCTGAACAACATGCATTCCGAG

RT-Gm7609-Fw	CAAGGAATGCGGGCCTTAC	RT-Enpp5-ex3-int3-Fw	TCTTGCTAACTTGGAGGCTGTGGA
RT-Gm7609-Rv	CCCCAAATACCAGCCAACAC	RT-Enpp5-ex3-int3-Rv	AAGTCTATGTGCCACCAAGCGGTA
RT-Xlr4b-Fw	CAGACCTCCCAAGCAACCA	RT-Enpp5-ex4-Fw	ACCTTCTCAGTTCAGCAACTCCCA
RT-Xlr4b-Rv	TGAAGCTGCTGTGAATCATTAGATG	RT-Enpp5-ex4-Rv	ATGATGCTGCCAAGAGAGACACCT
RT-Tmem116-Fw	ACCCTGCTGATCATGAAGCTGACA	RT-Inpp5f-ex23-Fw	TTGTGTCAGCACCTCGATTAGGCA
RT-Tmem116-Rv	TGCACTGTGTCCAGCCATAAATGC	RT-Inpp5f-ex23-Rv	TTCCCAAGTTCACTTCCAGGACCA
RT-Ccl25-Fw	GACTGCTGCCTGGGTTACCA	RT-Inpp5f-ex2-4-Fw	GCAGAAAGCGTTGGTAGGCAAACT
RT-Ccl25-Rv	CCTAGCATGCCGGAGAACA	RT-Inpp5f-ex2-4-Rv	AGCTCAAGCTCCTGAGGTTCCATT
RT-Pdlim3-Fw	GGCACCCCGAGTGCTTT	RT-App-ex6-7-Fw	TGATGATGAGGATGTGGAGGATGG
RT-Pdlim3-Rv	AGCCTTTCTGCTTGAGGTTGAG	RT-App-ex6-7-Rv	AAAGTACCAGCGGGAGATCATTGC
RT-Taf15-Fw	TCAAAACCAACAGTCTTACCATTCA	RT-App-ex10-11-Fw	CGTCAAGCCAAGAACTTGCCCAAA
RT-Taf15-Rv	CACGACGGTCATCTTGTGTGT	RT-App-ex10-11-Rv	TGTCTCTCATTGGCTGCTTCCTGT
RT-Adam15-Fw	TGGGCATGGCCATTCAG	RT-Gria1-ex15Fw	TGTGACACCATGAAAGTGGGAGGT
RT-Adam15-Rv	CATATTCACACCTCCGGAGAAGT	RT-Gria1-ex16Rv	GCCCTTGTCGTACCACCATTTGTT
RT-Pmm2-Fw	ACCTGCGGAAGGAGTTTGC	RT-Gria1-ex17Rv	TCAGCTTGTCTAAGACGCCTTGCT
RT-Pmm2-Rv	TGCTGATTTGGCCACCTATG	RT-Gria1-ex3-4Fw	GCCCTCCATGTGTGCTTCAT
RT-Hnrnpa1-Fw	GGGTTTGTCACATATGCCACTGT	RT-Gria1-ex3-4Rv	GGACAAACTGATTGGATGTGTCA
RT-Hnrnpa1-Rv	CCACCTTGTGTGGTCTTGCA	RT-Copg-3UTR1-Fw	CCTGGTGCTGCACACTTGTT
RT-Crtc3-Fw	TAACCCTTCCATCCAAGCCACACT	RT-Copg-3UTR1-Rv	GGCAGCCATCCTCCTGTTT
RT-Crtc3-Rv	TGGTGGAAAGAGACGGGTTGCTAA	RT-Copg-3UTR2-Fw	GTGCTCAGGGCCTCCACTT
RT-Gpsm3-Fw	CCCGGCTTCTTGAAGACAAA	RT-Copg-3UTR2-Rv	TGCACACGCTCCATTTGGT
RT-Gpsm3-Rv	CGCTGGCACTGGTGACTAAG	RT-Araf-3UTR1-Fw	TGGGATTGTGGGATCATTGG
RT-Bcs1l-Fw	CCGGGCGAGTAGATCTGAAG	RT-Araf-3UTR1-Rv	TCCCAGATGAAAAAAATGAAACC
RT-Bcs1l-Rv	CTGGGTCAGCTGCCAGTGT	RT-Araf-3UTR2-Fw	CTGTGAATGTAAGTGGGACAGGAT
RT-Nlgn1-Fw	TTCCAGCTGGGCTGTTAGTTTCCA	RT-Araf-3UTR2-Rv	CAGCATGGCTTTTTCCCAAA
RT-Nlgn1-Rv	TCACAGGTCCAAAGGCTATGTGGT	RT-Araf-3UTR3-Fw	CAGGGAGCCAATTTCAGCAT
RT-Tmem180-Fw	CATCACCTTGGGCCAGTACCT	RT-Araf-3UTR3-Rv	GGCAGAACAGTTATTGGCTGGTA
RT-Tmem180-Rv	CCCACGAACCACAGGAAGTT	RT-Araf-ex5-6-Fw	CAGTCACTGCCTGGGACACA
RT-Prrt2-Fw	TTGTGGCCTTCGCTTATGC	RT-Araf-ex5-6-Rv	CCACAATGAGCTCCTCACCAT
RT-Prrt2-Rv	CACGTCCCCCTGTTGCA	RT-Rnf14-ex3-4-Fw2	TCCTGCGCATTGCATTTTAC
RT-Gpr83-Fw	TGCTCGTGTGGCCAAGAA	RT-Rnf14-ex3-4-Rv2	GCCTGGTCTACAGAGCGAGTTC
RT-Gpr83-Rv	CTGTGGTCACGTCGCCAAT	RT-Rnf14-ex8-Fw	AAAAAGTTCAGAGAAGGGCAACA
RT-Ano2-Fw	GCTGTCACCTCCGACTTTATCC	RT-Rnf14-ex8-Rv	CCTCCCACACCAAGCTCTGT
RT-Ano2-Rv	TGTCCCGTTGTGGCTATAGGA	RT-Klhdc10-ex1-2-Fw	GGACAGCTCAACCGATTCGT
RT-Me3-Fw	CCCTGGAAAAGGAAGGCATAC	RT-Klhdc10-ex1-2-Rv	TCCCACCGTATTTTCTTCTTACCT
RT-Me3-Rv	TGGAGTCCACCATCCAGATCTT	RT-Klhdc10-ex4-6-Fw	CAACGGCAATGACGTCCAT
RT-Cd180-Fw	CTCTCCCACAGCCTCCTTGA	RT-Klhdc10-ex4-6-Rv	GACAGCTGAGTAGAGCCCATCTC

RT-Cd180-Rv	GCTGGCAGGCCATCGA	RT-Luc7l2-ex3-4-Fw	GCTGCCCTTGCTGACTCTTT
RT-Plekhn1-Fw	CTACGGGCCTCCTTTTCCA	RT-Luc7l2-ex3-4-Rv	ATGACCATGCTATGTGATCAACAA
RT-Plekhn1-Rv	TGGCGCTGTCTTCTCTGTTTC	RT-Luc7l2-ex6-7-Fw	TGTTGCCCCCACGATGTC
RT-Gp1bb-Fw	GCGCTGCTATTGGCACTTCTACTA	RT-Luc7l2-ex6-7-Rv	TGGACTTTCAAGCATTCTCCAA
RT-Gp1bb-Rv	AGAGAGAACTCTTGGATGGAGCGT	RT-Myh10-ex19-21-Fw	GCTTTGGAAGGACGAGATTCAG
RT-Xlr3b-Fw	CTGCTGGCAGGCACTCAA	RT-Myh10-ex19-21-Rv	TCATGAAGACCAGAAACACTGTCA
RT-Xlr3b-Rv	TGGGTTCTGGCTGTCATCTG	RT-Myh10-ex21-23-Fw	TCCGCTGCATCATTCCAA
RT-Celf3-Fw	GTCGAGGAGACCGGAAGCT	RT-Myh10-ex21-23-Rv	GGTGCGGGTCCAGTTTCC
RT-Celf3-Rv	CATCCTCATCTGTCTGCTGCTTT	RT-Abcc5-UTR1-Fw	GCTCCCAGTGTTCCATGCTT
RT-Pgap2-Fw	TGGCGGCTGACCAAGAA	RT-Abcc5-UTR1-Rv	CCAGGGAACTCGACAACTCTGT
RT-Pgap2-Rv	GCCGCTGTTTCCAGCTGTA	RT-Abcc5-UTR2-Fw	CAGCCACGTCTTGGCAGTACT
RT-Pspc1-Fw	AGCACCCGGACGAGGAA	RT-Abcc5-UTR2-Rv	GCTAGCCCGAGCCACACA
RT-Pspc1-Rv	CGGCTTGAGGAAACTCTTGATG	RT-Arntl-ex18-Fw	AAAGAGGACTCATCCCACTGTCCCA
RT-Atxn2l-Fw	TCCTGACAGCTGTAGTGGGTTCT	RT-Arntl-ex18-Rv	ATTTCCTCCGCGATCATTCGACCT
RT-Atxn2l-Rv	CCCTCGTAAGTGGTGCCATT	RT-Arntl-ex21-Fw	GCTTTGGCAACAGCTGCAGTATCA
RT-Chuk-Fw	GGCACAGTAACCCCTCCAGTAT	RT-Arntl-ex21-Rv	TGTATCAATGGCTCTGAGGTGGCT
RT-Chuk-Rv	CCACACATGTCAGAGGATGTTCA	RT-Gdap1l1-ex6dis-Fw	GTGGCACCCTAAAACCCATCT
RT-Tasp1-Fw	TCACCTTTCCTTGCCTGTGAA	RT-Gdap1l1-ex6dis-Rv	AGGGTGTGCCCATGGAATT
RT-Tasp1-Rv	CATGACCGGAGAACGATCACT	RT-Gdap1l1-ex6pro-Fw	ACCACTCTGCTGTCAGCTGTTATC
RT-Slc25a12-Fw	TCCTCCGAGACATTCCCTTCT	RT-Gdap1l1-ex6pro-Rv	TGGCGGTTTCCGTTTGA
RT-Slc25a12-Rv	GGAGTTTGCAGTGAGCGTACAC	RT-Tuba4a-ex5dis-Fw	GCCGCTTGTGACCTTTTAATTT
RT-Tac2-Fw	CCTGCTTCGGAGACTCTACGA	RT-Tuba4a-ex5dis-Rv	CCCCCTTGGCAAGTCTCA
RT-Tac2-Rv	GCACTTTCAGCAATCCTTCCA	RT-Tuba4a-ex5pro-Fw	GCGTGCTGTGTGCATGCT
RT-Zfp644-Fw	GGTGCTGACGAGGTTTACATTCT	RT-Tuba4a-ex5pro-Rv	ACGGGCCCAGGCTTCA
RT-Zfp644-Rv	ACAATGGTCCCCGAAAGACTAG	RT-Me3-ex9-10-Fw	GCTGAACTCTTGGCCAGAAGACAA
		RT-Me3-ex9-10-Rv	TGCCAACATCTAGCAGGACAGGAA

PCR-based detection of alternative RNA processing

For alternative splicing detection, primer sets were designed to amplify different isoforms in different sizes. The sequence of primers is listed above. PCR products were resolved by 1.0-2.5 % agarose gel electrophoresis or 5-20 % gradient polyacrylamide gel electrophoresis and stained with ethidium bromide or SYBR Gold (Molecular Probe). The fluorescence of PCR products was captured by LAS4000 (GE Healthcare). Intensity of band signals was quantified by Multi Gauge V3.1 software (FUJIFILM).

Histological analysis

Mice were anesthetized with pentobarbital and perfused through the left ventricle with 5 ml of PBS (pH7.4) to remove the blood followed by 50 ml of 4% paraformaldehyde in PBS (PH7.4). For preparation of paraffin blocks, the brains were collected, postfixed with the same fixative overnight and processed for paraffin embedding. The blocks were cut into 4-μm sections with cryostat. For frozen section preparation, the perfused brains were collected and further fixed with the same fixative overnight, followed by treatment with 20% sucrose in PBS for 2 days and then with 30% sucrose solution overnight. The brains were mounted in Tissue mount (Chiba Medical) and cut into 10 μm sections with a cryostat.

For immunohistochemistry, sections were treated with primary antibodies and detected using ABC Elite kit (Vector Laboratories) and diaminobenzidine substrate. When necessary, paraffin sections were pre-treated with autoclave in citrate buffer (pH 6.0) for antigen retrieval. For the analysis of skeletal muscles, frozen sections were prepared from quadriceps muscles of $TLS^{+/+}$ and $TLS^{-/-}$ mice and stained with HE. For counting the number of motor neurons, paraffin sections of thoracic spinal cord were stained with anti-choline acetyltransferase (Chat). For each animal, at least 9 sections (with intervals of at least 4 sections) were stained. The average number of Chat-positive cells was determined. Light microscopic images were captured using BIOREVO BZ-9000 (Keyence).

For counting Gems, hippocampal sections from 8 week old mice were stained with anti-SMN1. The number of nuclear dot-like staining of SMN1 was counted in each cell of the CA3 region from three animals of $TLS^{+/+}$ and $TLS^{-/-}$ mice. At least four microscopic fields per animal were used for counting.

Electron microscopy

8-12 week-old mice anesthetized with pentobarbital were perfused with 2% paraformaldehyde–2.5% glutaraldehyde in 0.1 M cacodylate buffer (pH 7.4). Brains were sectioned at 500 μm thickness, osmicated with 1% OsO_4 in cacodylate buffer, dehydrated through a gradient series of ethanol, and then embedded in epoxy resin (Epon 812, TAAB Laboratories Equipment Ltd., Berkshire, England). Ultrathin sections (80 nm thick) from the samples were cut with an ultramicrotome (Ultracut UCT, Leica Microsystems, Wetzlar,. Germany), collected on 200-mesh uncoated copper grids and then counterstained routinely with uranyl acetate and lead citrate. The sections were examined electron microscopically (Tecnai 12, FEI, Eindhoven, Netherlands).

Statistical analysis

We used a two-tailed unpaired t-test for comparison between two samples. We used ANOVA followed by Tukey's post-hoc test for multiple comparison. For the analysis of survival rate, we used the Kaplan-Meier method followed by log-rank test. We considered the differences significant when P value is less than 0.05. In bar charts and line charts, error bars represent standard errors of the mean. The number of samples and P values are described in the figure legends.

Supplemental References

1. Doi H, Mitsui K, Kurosawa M, Machida Y, Kuroiwa Y, Nukina N (2004) Identification of ubiquitin-interacting proteins in purified polyglutamine aggregates. FEBS Lett 571 (1-3):171-176.

2. Doi H, Okamura K, Bauer PO, Furukawa Y, Shimizu H, Kurosawa M, Machida Y, Miyazaki H, Mitsui K, Kuroiwa Y, Nukina N (2008) RNA-binding protein TLS is a major nuclear aggregate-interacting protein in huntingtin exon 1 with expanded polyglutamine-expressing cells. J Biol Chem 283 (10):6489-6500.

3. Emig D, Salomonis N, Baumbach J, Lengauer T, Conklin BR, Albrecht M (2010) AltAnalyze and DomainGraph: analyzing and visualizing exon expression data. Nucleic Acids Res 38 (Web Server issue):W755-762.

4. Hicks GG, Singh N, Nashabi A, Mai S, Bozek G, Klewes L, Arapovic D, White EK, Koury MJ, Oltz EM, Van Kaer L, Ruley HE (2000) Fus deficiency in mice results in defective B-lymphocyte development and activation, high levels of chromosomal instability and perinatal death. Nat Genet 24 (2):175-179.

5. Huang da W, Sherman BT, Lempicki RA (2009) Systematic and integrative analysis of large gene lists using DAVID bioinformatics resources. Nat Protoc 4 (1):44-57.

6. Kino Y, Washizu C, Oma Y, Onishi H, Nezu Y, Sasagawa N, Nukina N, Ishiura S (2009) MBNL and CELF proteins regulate alternative splicing of the skeletal muscle chloride channel CLCN1. Nucleic Acids Res 37 (19):6477-6490.

7. Lagier-Tourenne C, Polymenidou M, Hutt KR, Vu AQ, Baughn M, Huelga SC, Clutario KM, Ling SC, Liang TY, Mazur C, Wancewicz E, Kim AS, Watt A, Freier S, Hicks GG, Donohue JP, Shiue L, Bennett CF, Ravits J, Cleveland DW, Yeo GW (2012) Divergent

roles of ALS-linked proteins FUS/TLS and TDP-43 intersect in processing long pre-mRNAs. Nat Neurosci 15 (11):1488-1497.

8. Martin FC, Thu Le A, Handforth A (2005) Harmaline-induced tremor as a potential preclinical screening method for essential tremor medications. Mov Disord 20 (3):298-305.

9. Purdom E, Simpson KM, Robinson MD, Conboy JG, Lapuk AV, Speed TP (2008) FIRMA: a method for detection of alternative splicing from exon array data. Bioinformatics 24 (15):1707-1714.

10. Takahashi E, Niimi K, Itakura C (2011) Emotional behavior in heterozygous rolling mouse Nagoya Ca v 2.1 channel mutant mice. Neurobiol Aging 32 (3):486-496.

11. Yamashita Y, Matsuura T, Shinmi J, Amakusa Y, Masuda A, Ito M, Kinoshita M, Furuya H, Abe K, Ibi T, Sahashi K, Ohno K (2012) Four parameters increase the sensitivity and specificity of the exon array analysis and disclose 25 novel aberrantly spliced exons in myotonic dystrophy. J Hum Genet 57 (6):368-374.

Supplemental Tables (See separate Excel file)

Additional file 2: Table S1. Gene expression analysis of FUS/TLS-deficient mice.

Gene expression changes detected using ExonArray and AltAnalyze. Genes that were altered 1.2-fold at a raw P value of <0.005 are listed. Adjusted p values were calculated by the Benjamini-Hochberg method.

Additional file 3: Table S2. RNA processing analysis of FUS/TLS-deficient mice.

RNA processing changes detected using ExonArray and AltAnalyze. Exons that were altered by FIRMA fold change >2.0 at a raw P value of <0.005 are listed. Spread sheet modified from AltAnalyze.

Additional file 4: Table S3. Summary of validated RNA processing alterations in the TLS KO striatum.

Selected RNA processing events identified using AltAnalyze were validated either by qPCR or RT-PCR. The results are summarized. Statistical significance was analyzed by unpaired two-tailed t-test (n=3).

Additional file 5: Table S4. Transcriptome changes found in both this study and Lagier-Tourenne et al.

Changes in gene expression and RNA processing caused by FUS/TLS depletion in this study and a previous one are listed [7]. A relaxed threshold (P<0.05) was used for our list to maximize the detection of overlaps in these studies.

Kino et al. Figure.S1

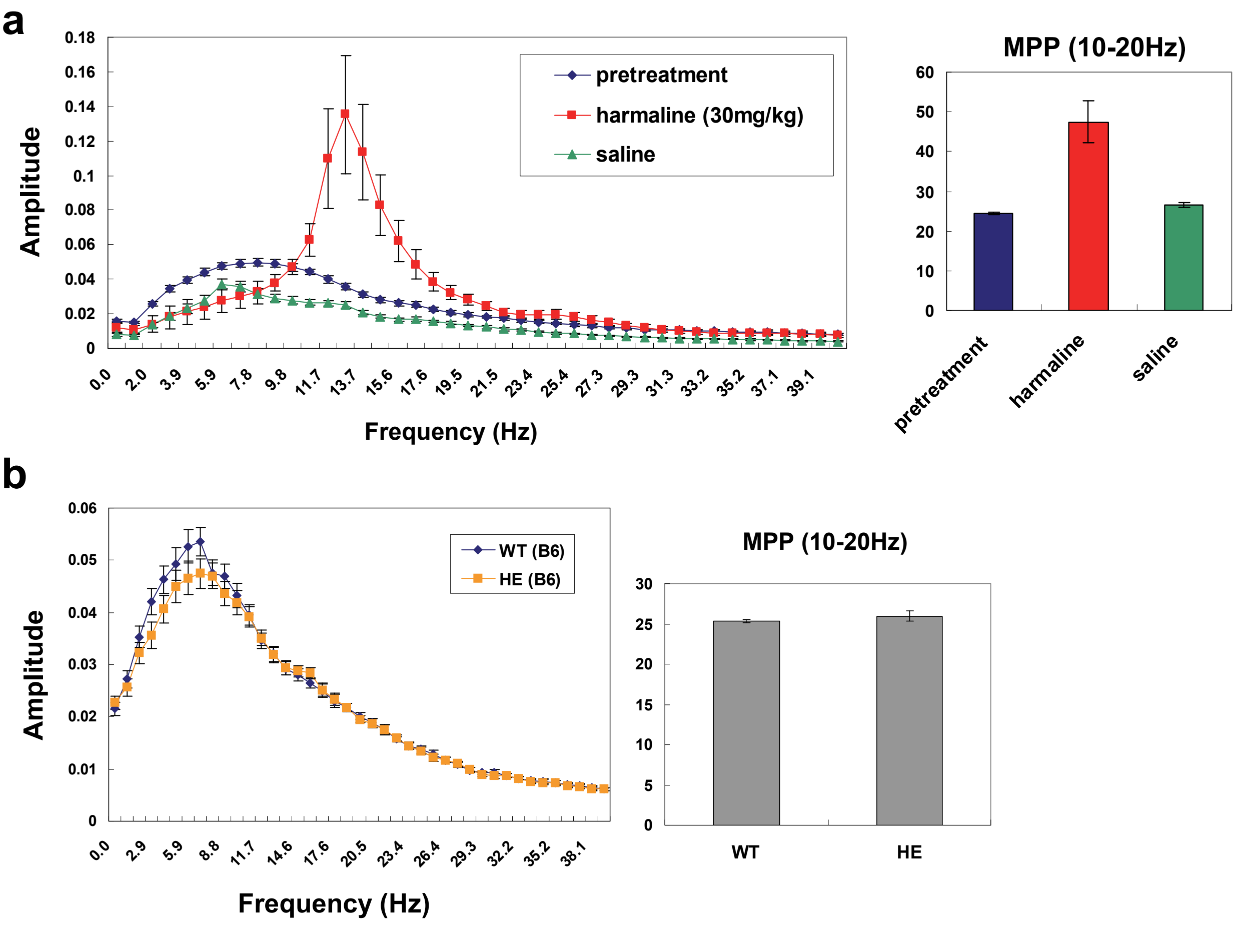

Figure S1

(a) Analysis of tremor-like movement of wild type mouse with or without harmaline treatment. Wild type mice of 8-12 weeks old were treated intraperitoneally with either saline or 30 mg/kg harmaline. Harmaline-treated mice clearly showed tremor-like movement starting from 5 minutes after treatment. Animal movement was recorded before and after injection. For the analysis of the drug effect, the data from 5 to 10 minutes after injection were used. Left chart shows the power spectra of animals with or without treatment (n=5 for saline and n=6 for harmaline treatment). Right chart shows the Motion power percentage (MPP) of these animals. **(b)** Analysis of tremor-like movement of inbred (B6) heterozygous FUS/TLS knockout mice and controls. Bar chart shows MPP analysis (mean ± SEM; WT, n=14; HE, n=10).

Kino et al. Figure.S2

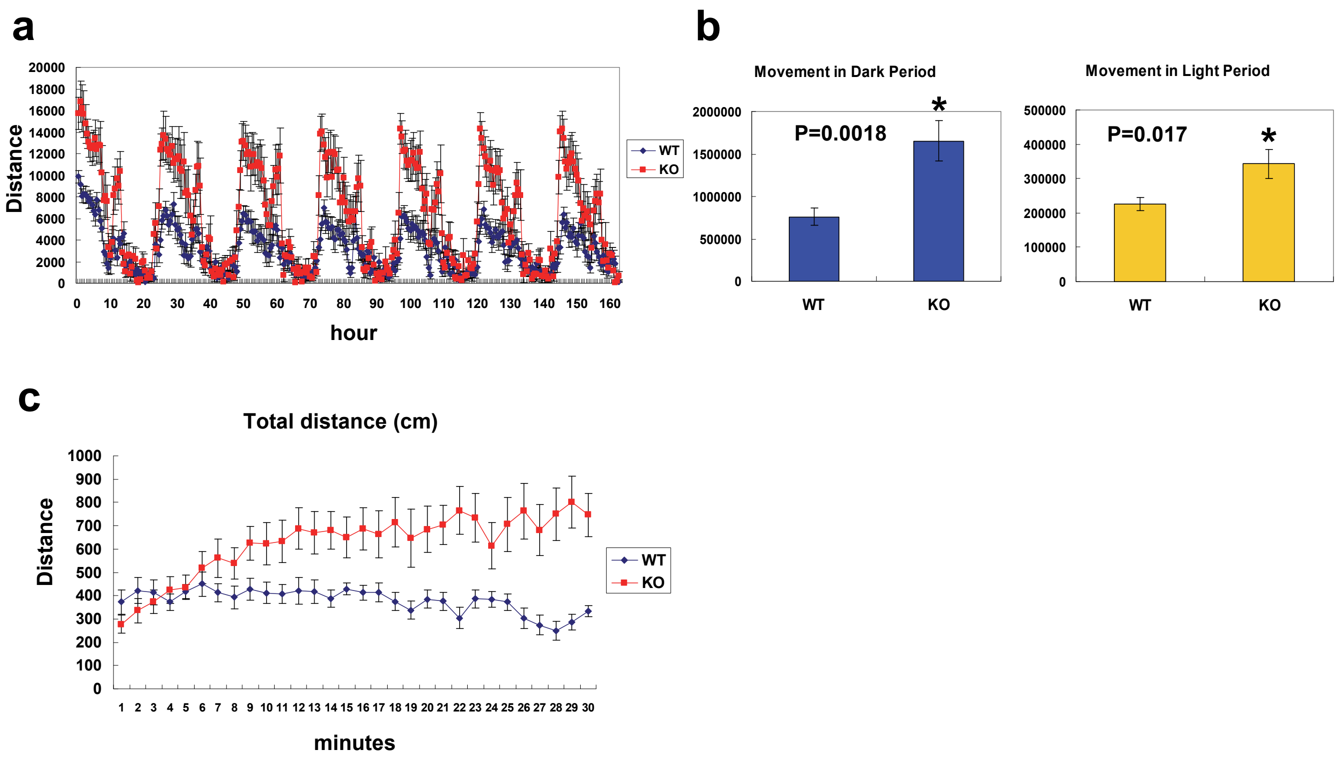

Figure S2

(a, b) Spontaneous home cage activity of WT and TLS$^{-/-}$ mice (the same date set as Figure 2A). Distance of movement are shown for each 30 minutes in (**a**). Movement of mice in dark and light periods were separetely analyzed in (**b**). P values were from unpaired two-tailed t-test (n=12 for WT, n=11 for KO animals). **(c)** Time course of locomotive activity in an open field test of FUS/TLS KO mice. Mean ± SEM (n=12 for WT, n=12 for KO). KO mice showed a time-dependent increase in locomotion.

Kino et al. Figure.S3

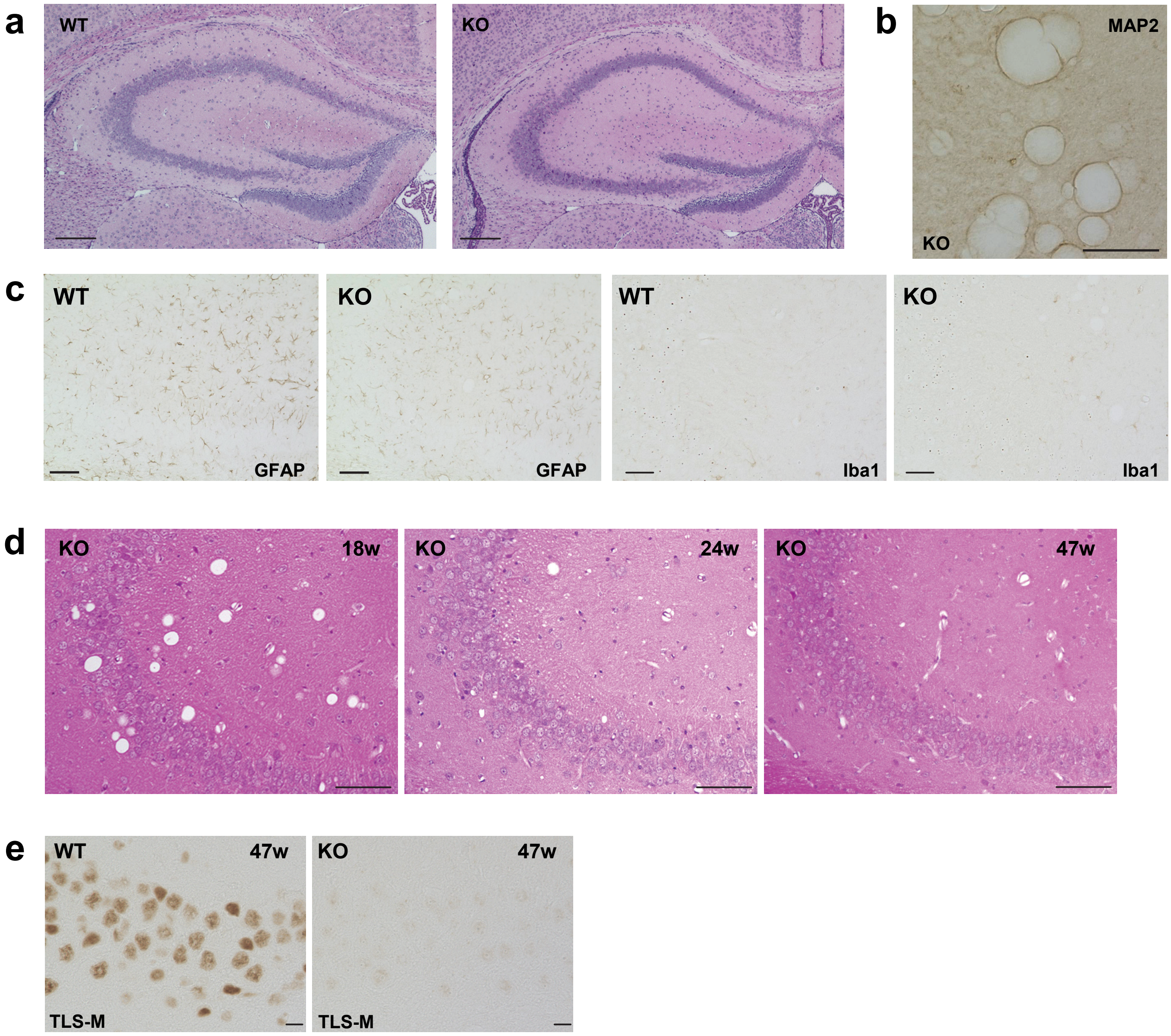

Figure S3

(a) Hippocampal sections of 3 week-old animals stained with hematoxylin-eosin. Scale bar: 200 μm. **(b)** MAP2 immunoreactivity around vacuole-like structures in the KO hippocampus . Scale bar: 50 μm. **(c)** Immunohistochemical analysis of GFAP and Iba1 in the hippocampus CA3 of TLS KO mice at 8 weeks. Scale bar: 50 μm. **(d)** Hippocampal sections of KO mice stained with HE. Shown are KO mice with (18 and 24 weeks) and without vacuolation (47 weeks). Scale bar = 100 μm. **(e)** TLS immunoreactivity in the CA3 region of WT and KO mice at 47 weeks. Scale bar = 10 μm.

Kino et al. Figure.S4

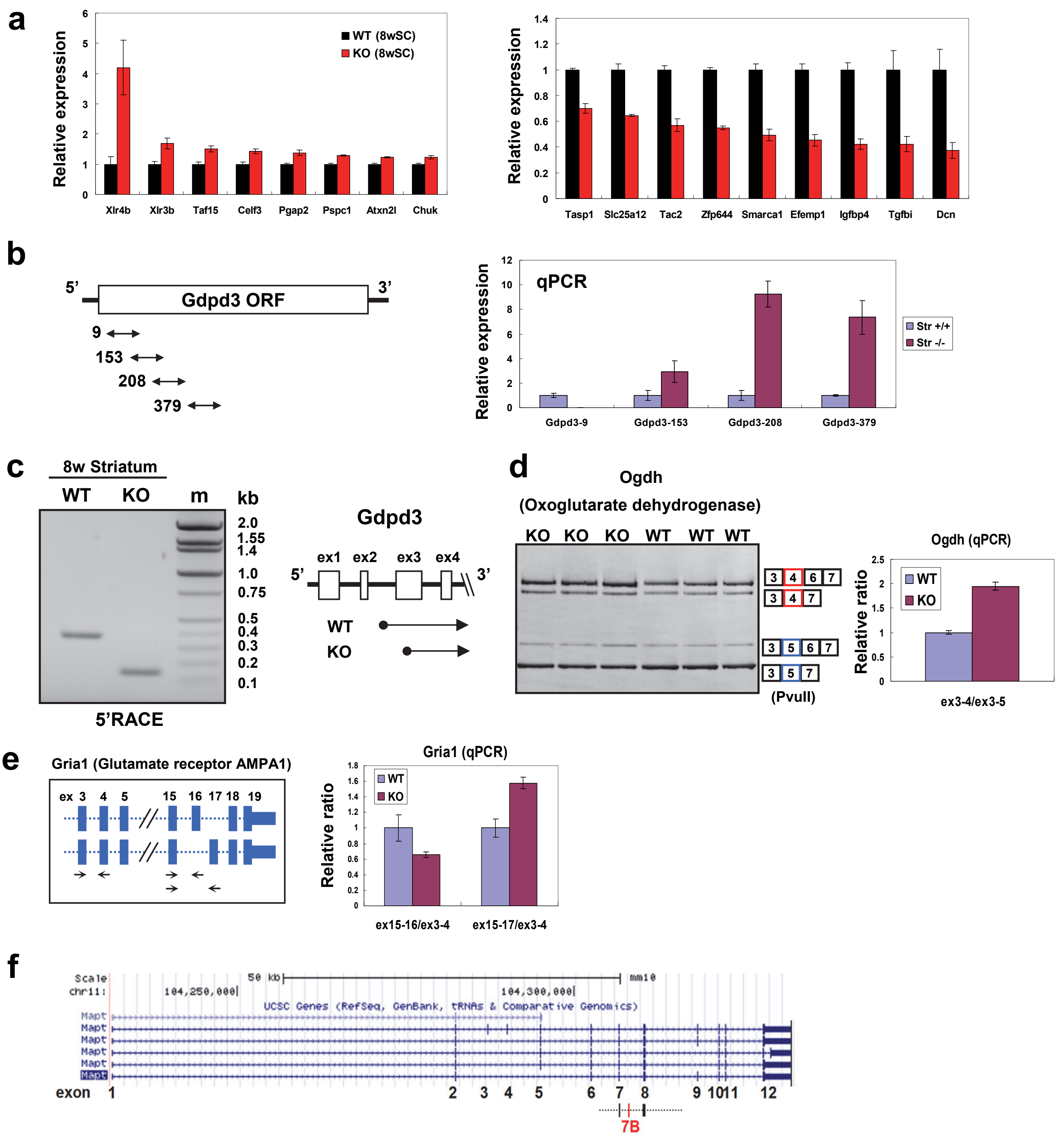

Figure S4

(a) Altered gene expression in the spinal cord of FUS/TLS KO mice at 8 weeks. qPCR results are shown (n=3) as in Fig. 4b. **(b)** Analysis of Gdpd3 expression in the striatum of TLS$^{-/-}$ and control mice. Right chart shows qPCR results using different primer sets (n=3). The locations of amplicons are indicated in the left. The expression of 5' region of Gdpd3 was smaller relative to the downstream region in TLS$^{-/-}$ mice. (**c**) Determination of the 5' end of Gdpd3 mRNA in WT and KO mice by 5'RACE. PCR products were resolved in agarose gel electrophoresis, excised, and sequenced. Gel image is shown in the right. 5' end of Gdpd3 in WT and KO mice. Both WT and KO mice produced 5'-truncated Gdpd3 mRNA, with more downstream transcriptional initiation in KO mice. (**d**) Alternative splicing of mutually exclusive exons 4 and 5 of Ogdh in TLS KO mice. RT-PCR and qPCR results are shown (n=3). (**e**) Alternative splicing of mutually exclusive exons 16 and 17 of Gria1. qPCR results using primer sets indicated in the upper panel are shown (n=3). (**f**) Gene structure of Mapt adopted from UCSC mouse genome browser. Exon numbers in this study are indicated. Exon 7B is a 54-nucleotide exon located between exons 7 and 8.

Kino et al. Figure.S5

a

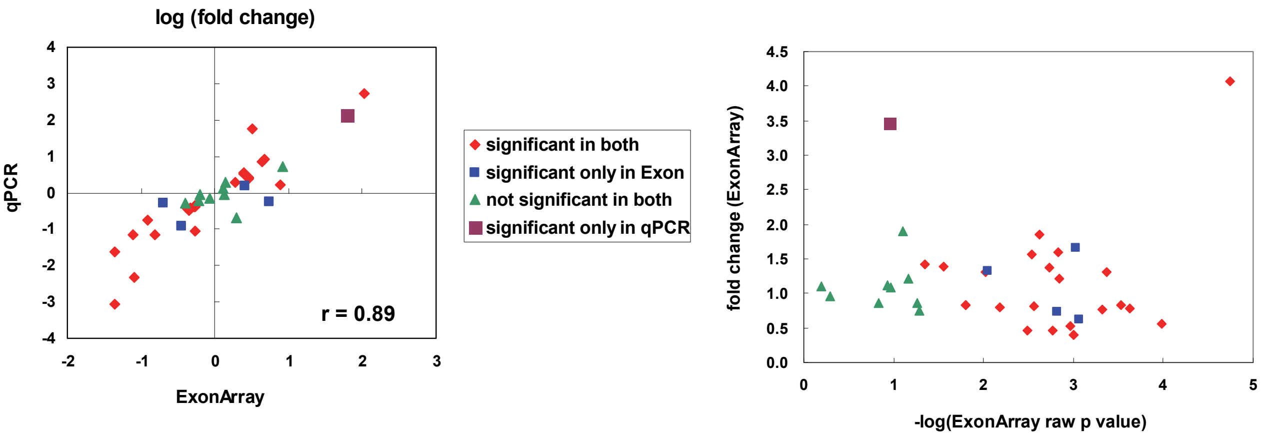

b

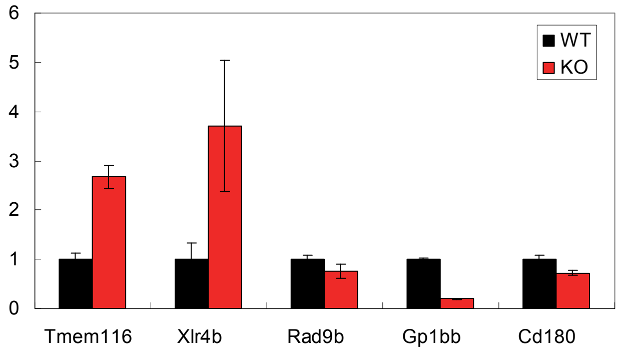

Figure S5

(a) Comparison of ExonArray and qPCR results (n=37 genes). Fold change of differentially expressed genes obtained by each method (log-transformed) were plotted (left panel). Genes were plotted according to their raw p values and fold change detected by ExonArray (right panel). Genes were classified into four groups depending on the significance and consistency of the results. See also Supplemental Materials and Methods (Microarray analysis). **(b)** Replication of qPCR results using additional sets of animals. cDNA samples from the striatum of WT and KO animals at 8 weeks were examined (mean+/-SE, n=3). We detected gene expression changes that were consistent with the initial sets of animals that are shown in Fig.4b.
